# Pan-cancer dissection of vasculogenic mimicry characteristic to provide potential therapeutic targets

**DOI:** 10.3389/fphar.2024.1346719

**Published:** 2024-04-17

**Authors:** Haibin Tang, Liuxun Chen, Xvdong Liu, Shengjie Zeng, Hao Tan, Gang Chen

**Affiliations:** ^1^ Department of Urology, The First Affiliated Hospital of Chongqing Medical University, Chongqing, China; ^2^ Department of Urology, The Second Affiliated Hospital of Chongqing Medical University, Chongqing, China; ^3^ Department of Cardiothoracic Surgery, The First Affiliated Hospital of Chongqing Medical University, Chongqing, China; ^4^ Department of Orthopedics, The First Affiliated Hospital of Chongqing Medical University, Chongqing, China

**Keywords:** vasculogenic mimicry, therapy target, immunotherapy, tumor microenvironment, immunosuppressive microenvironment, pan cancer analysis

## Abstract

**Introduction:**

Vasculogenic mimicry (VM) represents a novel form of tumor angiogenesis that is associated with tumor invasiveness and drug resistance. However, the VM landscape across cancer types remains poorly understood. In this study, we elucidate the characterizations of VM across cancers based on multi-omics data and provide potential targeted therapeutic strategies.

**Methods:**

Multi-omics data from The Cancer Genome Atlas was used to conduct comprehensive analyses of the characteristics of VM related genes (VRGs) across cancer types. Pan-cancer vasculogenic mimicry score was established to provide a depiction of the VM landscape across cancer types. The correlation between VM and cancer phenotypes was conducted to explore potential regulatory mechanisms of VM. We further systematically examined the relationship between VM and both tumor immunity and tumor microenvironment (TME). In addition, cell communication analysis based on single-cell transcriptome data was used to investigate the interactions between VM cells and TME. Finally, transcriptional and drug response data from the Genomics of Drug Sensitivity in Cancer database were utilized to identify potential therapeutic targets and drugs. The impact of VM on immunotherapy was also further clarified.

**Results:**

Our study revealed that VRGs were dysregulated in tumor and regulated by multiple mechanisms. Then, VM level was found to be heterogeneous among different tumors and correlated with tumor invasiveness, metastatic potential, malignancy, and prognosis. VM was found to be strongly associated with epithelial-mesenchymal transition (EMT). Further analyses revealed cancer-associated fibroblasts can promote EMT and VM formation. Furthermore, the immune-suppressive state is associated with a microenvironment characterized by high levels of VM. VM score can be used as an indicator to predict the effect of immunotherapy. Finally, seven potential drugs targeting VM were identified.

**Conclusion:**

In conclusion, we elucidate the characteristics and key regulatory mechanisms of VM across various cancer types, underscoring the pivotal role of CAFs in VM. VM was further found to be associated with the immunosuppressive TME. We also provide clues for the research of drugs targeting VM. Our study provides an initial overview and reference point for future research on VM, opening up new avenues for therapeutic intervention.

## Introduction

Sufficient blood supply is necessary to solid tumors growth, proliferation, survival, and development (Hanahan and Weinberg, 2011). Vasculogenic mimicry (VM) was firstly proposed in 1999, which refers to the ability of tumor cells to form their own blood vessels without relying on angiogenesis or the involvement of endothelial cells (Maniotis et al., 1999; Hendrix et al., 2003). These altered cells create a matrix-enriched tubular network that imitates the extracellular matrix of blood vessels (Wei et al., 2021). These channels provide an irrigation system for tumors to meet their metabolic and nutrient demands. VM is commonly observed in solid tumors such as such as hepatocellular carcinoma, melanoma, gastric cancer, colorectal cancer, lung cancer as well as breast cancer, and promotes tumor growth and metastasis by providing a blood supply (Luo et al., 2020). This phenomenon has been confirmed by multiple studies to be linked to decreased survival rates in cancer patients and associated with high tumor grade, progression, invasion, metastasis, and poor prognosis in patients with malignant tumors (Cao et al., 2013; Yang JP. et al., 2016; Lv et al., 2017; Ren et al., 2019). Moreover, the existence of VM also leads to the failure of anti-angiogenic therapy in tumor patients (Lin et al., 2023). Therefore, elucidating its characteristics will help to deepen the understanding of tumor aggressiveness and develop targeted drugs to improve patient prognosis.

VM is a complex and multifactorial process that involves various mechanisms, signaling pathways, and interactions with the tumor microenvironment (TME), and plays a crucial role in tumor progression and metastasis. Several potential mechanisms and signaling pathways, such as epithelial-mesenchymal transition (EMT), angiogenesis, hypoxia, WNT/β-catenin signaling, NOTCH signaling and cancer stem cells (CSCs) promote this process (Luo et al., 2020; Wei et al., 2021). Recent research suggests CSCs and EMT may be the main factors in the formation of VM(10). Cancer cells with VM competence often exhibit plasticity such that the cells have a dedifferentiated phenotype (Murai and Matsuda, 2023). EMT can cause tumor cells to lose epithelial markers and acquire mesenchymal characteristics, giving tumor cells phenotypic plasticity, thereby enhance the motility and invasiveness of tumor cells and facilitate VM formation (Xiao et al., 2023). In addition, various cytokines have been identified as contributing to the formation of VM, such as IL6, IL8, IL17, VEGF and TGF-β (Yang et al., 2016; Sharma et al., 2018; Bajbouj et al., 2022; Hu et al., 2022). Most of the them work by activating the biological process of EMT. Moreover, the interaction between VM and the TME has the potential to facilitate tumor progression (Luo et al., 2020; Wei et al., 2021; Hu et al., 2022). Previous studies have shown that tumor associated macrophages (TAMs) and cancer associated fibroblasts (CAFs) can interact with tumor cells and activate multiple signaling pathways, such as IL-6-JAK-STAT3, PI3K/AKT, ERK1/2 and VEGFA -165/Flt-1, thereby promoting the formation of functional VM channels (Luo et al., 2020; Barnett et al., 2016; Q et al., 2022; Tan et al., 2022; Pan et al., 2020; Ding et al., 2018; Kim et al., 2019; Liu et al., 2021; Wu et al., 2023). Although research on VM has made increasing progress, the mechanism by which VM occurs has not yet been fully elucidated. In addition, although previous studies have demonstrated the promoting effect of TAMs and CAFs on VM, relevant research is still limited. The relationship between VM and other components of the TME is still unclear. And the interaction between VM and various components in the TME remains to be further studied.

The development of multi-omics, high-throughput, and single-cell technologies has led to further exploration of the mechanisms of tumorigenesis and development and identification of clinical translational values. Here, we integrated multi-omics data from 21 epithelial-origin cancer types in TCGA along with single-cell RNA (scRNA) sequencing data from seven cancer types. We firstly characterized the molecular characterization of VM related genes (VRGs) in different cancer types. We the developed a pan-cancer VM score to provide a depiction of the VM landscape across cancer types, and to enable the comparison and stratification of tumors based on their VM level. The predictive ability of VM score on clinical prognosis was further verified. To further dissect VM, We then explored the potential regulatory mechanisms of VM by examining the correlation between VM and cancer phenotypes. Our results demonstrate that universal patterns of VM transcend different cancer types and that intratumoral VM levels are precisely controlled by multiple types of biological processes. In addition, the impact of VM on the TME was also evaluated. Our study particularly emphasized that VM shapes an immunosuppressive microenvironment to promote tumor immune evasion. Moreover, we also provide potential therapeutic drugs for targeted VM in clinical practice and construct a predictive model to assess patient prognosis. To the best of our knowledge, we are the first to elucidate the characterization of VM across cancers based on multi-omics data. Moreover, we performed a cell communication analysis based on single-cell data to investigate the interactions between VM cells and TME cells, which is a novel and innovative approach to study the interaction of VM cell with microenvironment. Our study provides an initial overview and reference point for future research on VM to understand how this unique feature can be exploited to improve outcomes for cancer patients.

## Methods

### Data collection

The transcripts per million (TPM) mRNA sequencing and associated clinical data generated by The Cancer Genome Atlas (TCGA) for 32 cancer types were obtained from GDC Data Portal (https://portal.gdc.cancer.gov/). 21 epithelial tumors with tumor samples greater than 100 were used for subsequent analysis (Supplementary Table S1). DNA methylation data (Methylation450K), copy number variation (CNV) and DNA methylation based stemness scores (DNAss) of TCGA samples were downloaded from UCSC Xena portal (https://xenabrowser.net/). The gene set of VM comprising 36 genes was collected from previous studies (Supplementary Table S2). The 50 cancer Hallmark gene sets were obtained from the Molecular Signatures Database (MSigDB, http://www.gsea-msigdb.org/gsea/msigdb). Single cell RNA (scRNA) sequencing datasets and related cell annotation information of seven different cancer types including BRCA, PAAD, STAD, HNSC, NSCLC, KIRC, LIHC were downloaded from TISCH portal (http://tisch.comp-genomics.org/) (Sun et al., 2021). The scRNA sequencing data of KIRC including 19 tumor samples were downloaded from the Gene Expression Omnibus (GEO) database with accession number GSE207493 (Yu et al., 2023). The expression and clinical response data for the immunotherapy cohort were obtained from Supplementary Material of Braun DA, Hou Y, Bakouny Z, et al. article, and GEO database with accession number GSE78220, GSE135222 and GSE100797, respectively (Lauss et al., 2017; Taylor et al., 2018; Jung et al., 2019; Braun et al., 2020). The mRNA sequencing and associated clinical data of E-MTAB-1980 cohort was obtained from EMBL-EBI database (https://www.ebi.ac.uk/). The above contents are summarized in Table 1.

**TABLE 1:** Data sources used in this article.

Data set	Database	Data type	Detailed information
Pancer cancer cohort	TCGA	TPM mRNA sequencing and clinical data	21 epithelial tumors
Pancer cancer cohort	UCSC	DNA methylation data, copy number variation data, DNA methylation based stemness scores	21 epithelial tumors
Pancer cancer cohort	TISCH	scRNA sequencing and cell annotation data	7 cancer types including: BRCA, KIRC, LIHC, NSCLC, OV, PAAD, STAD
E-MTAB-1980	EMBL-EBI	mRNA sequencing and clinical data	101 KIRC patients
GSE207493	GEO	scRNA sequencing data	19 KIRC samples
GSE78220	GEO	Expression and clinical response for immunotherapy	28 melanoma patients with PD-1 checkpoint inhibition therapy
GSE135222	GEO	Expression and clinical response for immunotherapy	27 NSCLC patients with anti-PD-1/PD-L1 therapy
GSE100797	GEO	Expression and clinical response for immunotherapy	25 melanoma patients with T-cell therapy
PMCID: PMC7499153	-	Expression and clinical response for immunotherapy	311 KIRC patients with PD-1 checkpoint inhibition

BRCA, breast cancer; KIRC, kidney renal clear cell carcinoma; LIHC, liver hepatocellular carcinoma; NSCLC, non-small cell lung cancer; OV, ovarian serous cystadenocarcinoma; PAAD, pancreatic adenocarcinoma; STAD, stomach adenocarcinoma.

### Analysis of vasculogenic mimicry profile across cancers

Among 21 tumor types, 15 tumor types included more than five pairs of tumor and normal samples were selected to explore expression differences in the vasculogenic mimicry profiles between tumor and adjacent normal tissue by using R package ‘limma’ with default parameters (Ritchie et al., 2015). The potential protein–protein interaction (PPI) network of the VRGs was constructed by using the Search Tool for the Retrieval of Interacting Genes Database (STRING) database (https://string-db.org/) and visualized by using Cytoscape (version 3.9.0) software (Shannon et al., 2003). Genomic alterations of vasculogenic mimicry associated genes were then explored at pan-cancer level.

### Genomic variation and methylation analysis

The gene-level CNVs obtained from UCSC Xena portal was estimated by using the GISTIC 2.0 threshold method (Mermel et al., 2011). The estimated thresholds are set to −2, −1, 0, 1, 2, representing homozygous deletion, single copy deletion, diploid normal copy, low-level copy number amplification, or high-level copy number amplification, respectively. We consider thresholds equal to 2 as amplifications, and thresholds equal to −2 as deep deletions according to previous study (Beroukhim et al., 2010).

Tumor mutation burden (TMB) of each sample were calculated as the number of non-synonymous somatic mutations per megabase according to the methods in previous publication (Lv et al., 2020). Somatic mutations data of pancancer were further analyzed and visualized by using R package ‘maftools’ with default parameter settings (Mayakonda et al., 2018).

Pearson correlation analysis was performed to evaluate the relationship between methylation levels and expression of VRGs by using the DNA methylation data downloaded from UCSC Xena.

### Evaluation of vasculogenic mimicry score

Single-sample gene-set enrichment analysis (ssGSEA) can represent the extent to which genes in a specific gene set are coherently increased or decreased within a sample by calculating a separate enrichment score, which reflects the activity level of the biological process or pathway associated with the gene set in each sample (Barbie et al., 2009). To further investigate the potential biological function of VM from the level of pathway activity, ssGSEA was applied to quantify the enrichment score of vasculogenic mimicry and Hallmark pathways in each individual TCGA sample by using ‘GSVA’ R package with default parameters (Hänzelmann et al., 2013). The enrichment score of VM for each sample were defined as the VM score. The difference of VM score between tumor and normal samples in each tumor was estimated by applying the ‘ggpubr’ R package (Wickham, 2016). Then, Pearson correlation analysis was performed between ssGSEA enrichment score of each Hallmark gene sets and VM to explore the potential biological function of VM. R package ‘pheatmap’ was used for visualizing.

### Survival analysis

R package ‘survival’ (https://github.com/therneau/survival) was used to perform survival analysis. The ‘Survminer’ R package (https://github.com/kassambara/survminer) was applied to screen the optimal cut-off point of VM score to stratify patients in each cancer type into high- and low-groups. The single variate COX regression analysis, Kaplan-Meier survival curve, and log-rank tests were then conducted to evaluate the prognostic difference between the high- and low-VM score groups. Patients in the pan-cancer dataset were stratified into four groups according to quartiles of VM score. The pancancer Kaplan–Meier survival curve was also plotted to evaluate the overall effect of VM on survival.

### Tumor microenvironment and immune infiltrating analysis

Cell contents in each sample microenvironment were inferred by using six different algorithms, including CIBERSORT, cellreport, xCell, QUANTISEq, MCPcount and EPIC (Newman et al., 2015; Becht et al., 2016; Aran et al., 2017; Charoentong et al., 2017; Racle et al., 2017; Finotello et al., 2019). CIBERSORT, xCell, QUANTISEq, MCPcount and EPIC are five algorithms that use deconvolution to estimate immune infiltration in the TME from gene expression data. Cellreport takes a set of gene sets that represent specific immune cell subpopulations and uses gene set enrichment analysis (GSEA) to calculate the microenvironmental composition and cell densities (Charoentong et al., 2017). Different algorithms are calculated based on different cell expression profiles and include different immune cell states. Characterizing the TME through multiple algorithms can more accurately and comprehensively reflect the characteristics of the TME. R package ‘complexHeatmap’ was used to illustrate the infiltration levels of different cellular components in the microenvironment between high and low VM score groups (Gu et al., 2016; Gu, 2022). Estimation of STromal and Immune cells in MAlignant Tumor tissues using Expression data (ESTIMATE) algorithm was applied to calculate the immuneScore (representing immune cell infiltration level), stromalScore (representing stromal cell infiltration level) and ESTIMATEScore (negatively correlated with tumor purity) for each cancer patients. Pearson correlation analysis was applied evaluate to correlations between VM score and these three scores for each cancer type (Yoshihara et al., 2013). Tumor immune dysfunction and exclusion (TIDE) (http://tide.dfci.harvard.edu/) was used to compute immune dysfunction, immune exclusion scores, CAFs of TME at pan-cancer level. The TIDE score which can serve as a surrogate biomarker to predict the response to immune checkpoint blockade was also calculated (Jiang et al., 2018). In addition, immunotherapy effect prediction data are also obtained from TIDE. The activity of tumor-infiltrating immune cells in the seven-step cancer immune cycle was also obtained from the http://biocc.hrbmu.edu.cn/TIP/to further analyze the immune characteristics of the microenvironment (Xu et al., 2018).

### Drug sensitivity analysis

The gene expression profile of 809 tumor cell lines and corresponding response data of 198 compounds of each cell lines were downloaded from the Genomicsof Drug Sensitivity in Cancer (GDSC) (https://www.cancerrxgene.org/) dataset (Yang et al., 2013). VM score of each cell was also calculated by using ssGSEA method. Pearson correlation analysis was then conducted to explore drugs that potentially associated with vasculogenic mimicry.

### scRNA-seq data analysis

The R package ‘Seurat’ was applied for quality control (QC) and downstream analysis (Hao et al., 2021). Seurat objects of seven individual cancer types including STAD, KIRC, NSCLC, LIHC, BRCA, PAAD were firstly generated. The function merge was used to merge Seurat objects of different cancers. Non-negative matrix factorization (NMF) is a matrix factorization method that can reduce the dimension of expression data from thousands of genes to a handful of metagenes in single-cell transcriptomic data. Based on the cell type annotation results, we extracted the malignant cells for NMF analysis to explore distinct molecular patterns in VM cells by using ‘NMF’ R package (http://renozao.github.io/NMF/). First, the number of modules for each tumor is obtained based on the optimal factor of the similarity matrix. Scores in each module for all VM cells were calculated by using AddModuleScore function. Next, Pearson correlation analysis was used to calculate the correlation between modules to explore similar gene programs among tumors. The top scoring 30 genes in each program were considered to represent the characteristics of the module.

The Seurat object of 19 KIRC samples was generated with the filter criteria of cells with less than 500 genes detected and the number of genes more than twice of the median number of detected genes (potential doublets). After data normalization, the top 3,000 highly variable genes were identified by using FindVariable function. The R package ‘Harmony’ was used to eliminate the batch effect between different cancer samples (Korsunsky et al., 2019). We performed cell clustering based on the top 30 principal components (PCs) and dimension reduction using uniform manifold approximation and projection (UMAP). Function AddModuleScore was conducted to calculate VM score of tumor cells. ‘CellChat’ R package was applied to analyze and visualize cell interaction communication networks in the TME (Jin et al., 2021). ‘pySCENIC’ package (https://github.com/aertslab/pySCENIC) in python was conducted to infer transcription factors that potentially regulate VM with default parameters. ‘SCENIC’ package was applied to infer VM cell-specific transcription factors (Aibar et al., 2017). Unless otherwise stated, all functions and algorithms described above use default parameters.

### Construction of VM related prognostic models based on multiple machine learning algorithms

Univariate COX regression analysis is used to determine potential prognostic markers in VRGs. Then, 10 machine learning algorithms such as random forest survival (RSF), elastic network (Enet), Lasso, Ridge, stepwise Cox, CoxBoost, Cox least squares regression (plsRcox), principal component analysis (SuperPC), generalized additive regression model (GBM) and survival support vector machine (SVM) were combined into 101 algorithms to establish a stable prognostic model. The combined machine learning algorithms are similar to previous studies, and detailed information about the functions and parameters of the scripts can be found in the GitHub website (https://github.com/Zaoqu-Liu/IRLS) (Liu Z. et al., 2022; Li et al., 2023). In our study, TCGA cohort is set as the training cohort, E-MTAB-1980 cohort as the test cohort. Harrell concordance index (C-index) was calculated for each cohort. The optimal model is defined as a model that has a high C-index in both training and validation cohorts. Each patient’s VM related score (VRS) was calculated based on the expression of genes and their corresponding coefficients. In each cohort, patients were divided into high VRS group and low VRS group by using median VRS as the cutoff value. Univariate and Multivariate Cox analysis were used to determine predictive value of VRS. Finally, the predictive value of VRS immunotherapy was validated in an KIRC immunotherapy treatment cohort.

### Statistical analysis

R (version 4.1.1) was used for all statistical analyses in the study except the pySCENIC analysis. pySCENIC was conducted by using Python (3.12.0). Student’s t-test or Wilcoxon test was used for comparisons of continuous variables between groups. The Kruskal–Wallis test was used for comparisons of multiple groups of continuous variables. Survival differences between different groups were determined by log-rank test. *p*-value less than 0.05 was considered statistically significant.

## Results

### Expression, Somatic alteration, methylation and interactions of vasculogenic mimicry genes in pan-cancer

To understand the expression, somatic alteration and interaction of the 36 VRGs at the pan-cancer level, the mRNA profile, SNP, CNV and methylation data of TCGA samples were obtained for following analyses.

**FIGURE 1:**
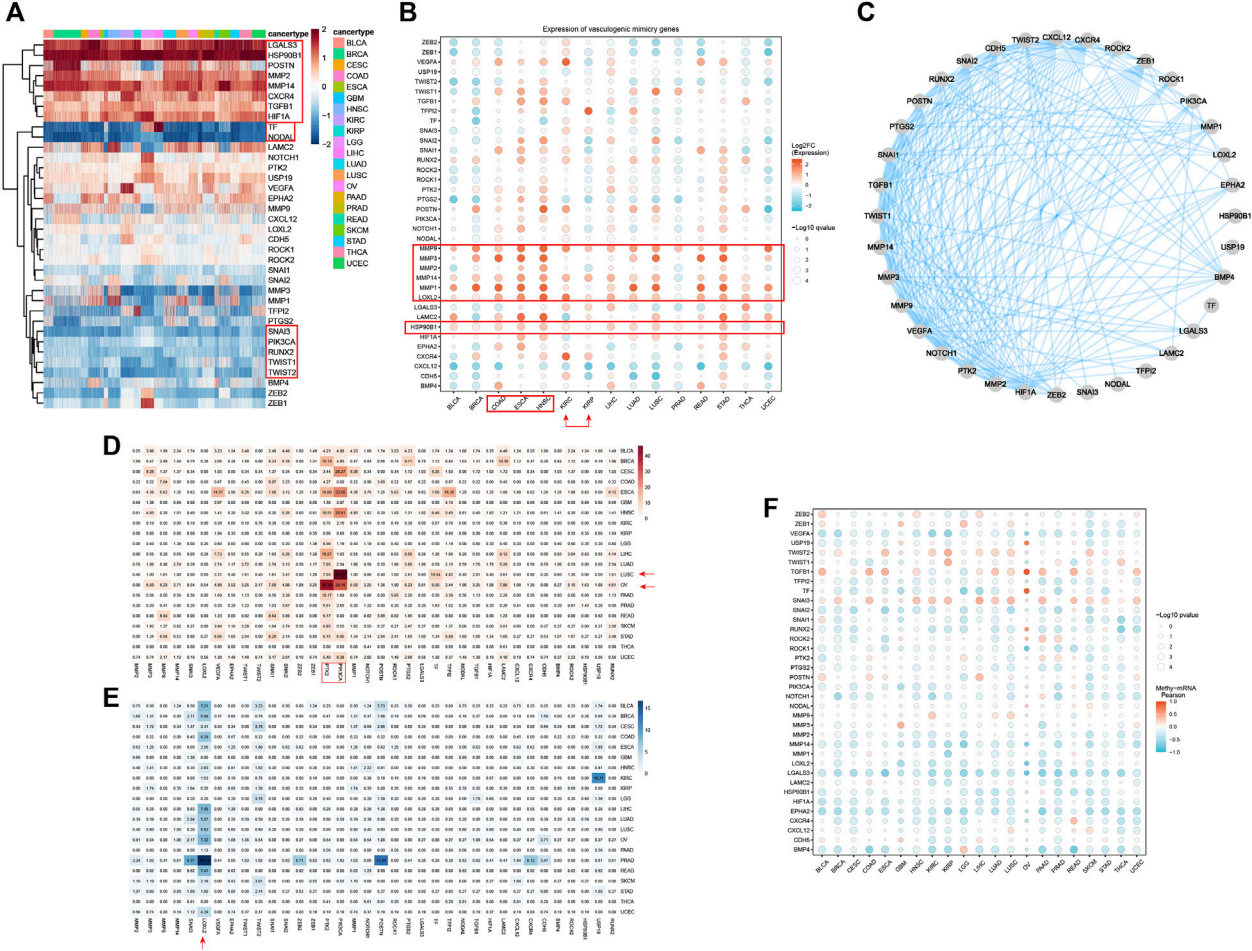
Expression, Somatic alteration, methylation and interactions of vasculogenic mimicry genes (VRGs). **(A)** Expression patterns of VRGs in pan-cancer. **(B)** Expression changes of VRGs in tumor tissue and para-tumor tissue in different tumor types. **(C)** Protein–protein interactions network of VRGs. **(D)** The amplification ratio of VRGs in different tumor types. **(E)** The deletion proportion VRGs in different tumor types. **(F)** The relationship between the expression and methylation levels of vascular mimicry-related genes in different tumor types.

The distribution expression of 36 VRGs was shown in Figure 1A. Out of these 36 genes, LGALS3, HSP90B1, MMP2, MMP14, CXCR4, TGFB1, HIF1A were observed significantly overexpressed in all cancer types, especially LGALS3, HSP90B1. While the expression levels of TF, NODAL, SNAI3, PIK3CA, RUNX2, TWIST1, TWIST2 remain low. The expression of PTK2, USP19, LAMC2, VEGFA, EPHA2, and MMP2 varies greatly among different cancer samples and has relatively high expression. Differential expression analysis between tumor and adjacent normal tissues in each individual cancer types was then conducted to exhibit the dysregulation patterns of VRGs (Figure 1B). Similar to expression profiles, MMP14, MMP1, LOXL2, MMP9, HSP90B1 are the most upregulated in different types of tumors (Figure 1B; Supplementary Figure S1A). Notably, STAD, HNSC, LIHC, KIRC, ESCA and COAD, exhibited the highest accumulation of upregulated VRGs (Figures 1A,B; Supplementary Figure S1B). Although both KIRC and KIRP are originated from kidney tissue, their gene dysregulation profiles vary widely, reflecting heterogeneity among tumors of the same tissue (Figure 1B; Supplementary Figure S1C). Potential protein–protein interaction among VRGs were further explored by using the STRING database, which demonstrates VM is a complexly co-regulatory process involving a series of genetic interactions (Figure 1C).

Having investigated the transcriptome landscape of VRGs among different cancer types, we explored whether variation of VRGs could be explained from the aspects of genomics and epigenetics. We firstly analyzed the CNV data of VRGs to characterize their genomic changes within different cancer types. As shown in Figures 1D,F, most of the genes related to VM were mainly amplified. OV and LUSC had the highest amplification frequency of VRGs, especially for OV, which had the most genes amplified. PTK2 and PIK3CA were the most amplified genes in different tumors. PTK2 was generally amplified among different tumors, with highest amplification frequency in OV. PIK3CA was highly amplified mainly in CESC, ESCA, HNSC, LUSC, OV and was most frequently amplified in LUSC. VEGFA, SNAIL1, SNAIL1, MMP9, PTGS2, LAMC2, TF, TFPI2 also have a certain amplification in different tumors (Figure 1D). LOXL2 was the gene with the highest deletion frequency (Figure 1E). The deletion frequency of POSTN in PRAD is as high as 12.04%, while the deletion frequency of USP19 in KIRC is 10.71%. We further analyzed the relationship between gene CNV and expression. Supplementary Figure S1C shows that significantly higher gene expression in samples exhibiting amplification than in non-mutated and deletion samples, which suggests that CNV affects gene expression levels. Somatic mutation analysis showed that PIK3A was the gene with the highest mutation frequency, and missense mutations were dominant (Supplementary Figure S1D).

Subsequently, we explored the relationship between gene expression and gene methylation to identify epigenetic regulation at pan-cancer level. The results reflected the heterogeneity of methylation levels among different tumor types. LGG had the highest level of methylation. Among 36 genes, MMP1, HSP90B1, PTK2, MMP3, NOTCH1 and CDH5 had higher methylation status at pan-cancer level (Supplementary Figure S1E). Correlation analysis results suggest that the expression levels of most VRGs are negatively correlated with methylation levels. Only SNAI3, TGFB1, TWIST1, and TWIST2 are mainly positively correlated with methylation levels (Figure 1F). While their frequency of CNV is not high, which indicates that their expression may be regulated by more complex mechanisms. Our results show the dysregulation pattern of VRGs in different tumors, which is significantly heterogeneous. We also suggest that multiple regulatory mechanisms are involved in the regulation of VRGs expression, and CNV and methylation may be important regulatory mechanisms.

### Construction and characterization of vasculogenic mimicry score in pan-cancer level

**FIGURE 2:**
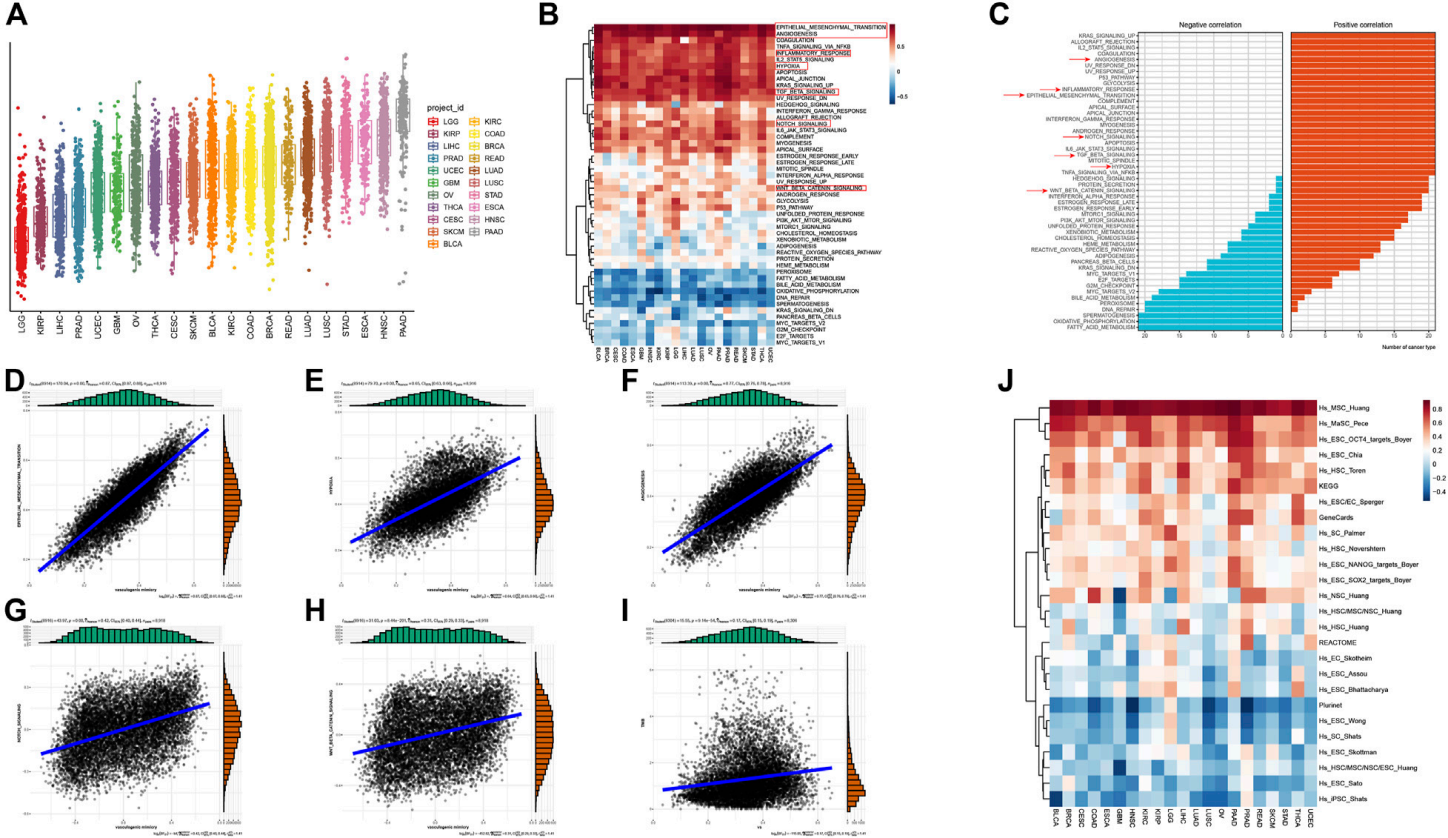
Construction and characterization of vasculogenic mimicry score (VM score) at pan-cancer level **(A)** pan-cancer distribution of VM score. **(B)** Correlation of VM level and 50 clearly defined Hallmark in different tumor types. **(C)** Summary of the correlation between different Hallmark and VM. **(D–I)** VM was associated with epithelial-mesenchymal transition, hypoxia, angiogenesis, NOTCH signaling pathway, WNT/β-catenin signaling pathway, and tumor mutation burden in pan-cancer. **(J)** VM level is positively correlated with cell stemness in pan-cancer.

To enable quantification of VM levels and evaluate the heterogeneity of VM activity among different types of cancer, enrichment score was calculated by ssGSEA. As shown in Figure 2A, the VM score varied widely across different cancer types and even within the same cancer. PAAD, HNSC, STAD, ESCA, LUSC exhibited relatively higher VM score, which are consistent with the expression level of VRGs. While the scores of LGG were the lowest, which is consistent with the findings that the methylation level of VRGs in LGG is the highest in the previous study (Supplementary Figure S1E). Interestingly, although KIRC and KIRP are both derived from kidney, their enrichment scores vary greatly. Similar results were also observed between LGG and GBM, LUSC and LUAD. These results demonstrated highly inter-tumor and intratumor heterogeneous VM status, which profoundly affects the prognosis and treatment outcomes of cancer patients (Dentro et al., 2021).

Given the abnormal activity of VM among various typed of cancer, we aimed to further dissect the underlying mechanisms of vasculogenic mimicry and its biological effects on association with tumor phenotypes, we further explored the relationship between VM and 50 cancer Hallmarks at pan-cancer level. VM is highly correlated with tumor associated pathways such as EMT, angiogenesis, hypoxia, WNT/β-catenin and NOTCH signaling (Figures 2B,C). Herein, VM was found to be the most strongly associated with EMT (R = 0.87, *p* < 0.001) (Figure 2D). EMT has been repeatedly reported to play a key role in VM formation (Qi et al., 2014; Yang et al., 2015; Luo et al., 2020; Kang et al., 2021). Strong association between VM and hypoxia (R = 0.65, *p* < 0.001), angiogenesis (R = 0.77, *p* < 0.001), NOTCH (R = 0.42, *p* < 0.001), WNT/β-catenin (R = 0.31, *p* < 0.001) were also exhibited (Figures 2E–H). It is notable that EMT, angiogenesis, hypoxia and NOTCH signaling were positively correlated with VM in all tumors (Figure 2C). WNT/β-catenin and NOTCH signaling pathways were confirmed to be involved in the process of EMT and VM in (Benjakul et al., 2022; Yuan et al., 2022). Cancer hallmarks such as P53 signaling, glycolysis, KRAS signaling pathways, and immune-related pathways such as TNF-α, TGF-β, IL2/STAT5, IL-6/JAK/STAT3, interferon-γ response, and inflammatory response pathways were also positively associated with VM (Figures 2B,C). Prior studies have suggested that immune response-related pathways can modulate VM formation by influencing the expression of VM related genes genes and activity of VM related genes proteins (Yang et al., 2015; Yang et al., 2016; Luo et al., 2020; Pan et al., 2020; Hu et al., 2022). TMB can indirectly reflect the ability and degree of neoantigen production by tumors. Further analysis showed that there was a positive correlation between the VM score and TMB (Figure 2I). In addition, we explore the relationship between VM and stemness characteristics of cancer samples (Figure 2J; Supplementary Figure S2). These results illustrated that VM is highly correlated with tumor malignancy and tumors with VM may activate multiple tumorigenic pathways, which may indicate a poor prognosis.

### Vasculogenic mimicry is associated with unfavorable tumor phenotypes and clinical outcomes

**FIGURE 3:**
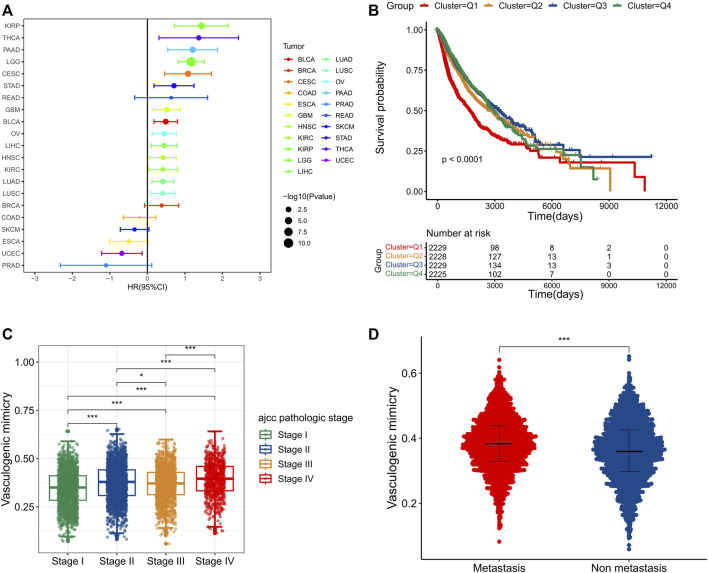
Vasculogenic mimicry (VM) is associated with unfavorable tumor phenotypes and clinical outcomes **(A)** A forest plot showing the hazard ratio of VM score in different cancer types. **(B)** A pan-cancer Kaplan–Meier curve shows survival associated with quantile stratified VM score. The log-rank test was used for the test of survival differences between all four groups. **(C)** A pan-cancer box plot shows that VM score is associated with tumor stages. The higher the tumor stages, the higher the VM score. **(D)** The box plot shows higher VM score in metastatic tumors. *p < 0.05; **p < 0.01; ***p < 0.001.

Subsequently, the impacts of VM on survival were further decoded to reveal the clinical relevance of VM. We first assessed the prognostic impact of VM score in each cancer type individually. Patients of each cancer type were stratified into two groups based on the cutoff determined by the ‘Survminer’ R package. The results of single variate Cox analysis and log-rank test demonstrated that the prognostic impact of VM varied between different cancers. In most cancer types, including KIRP, THCA, PAAD, LGG, CESC, STAD, READ, GBM, BLCA, OV, LIHC, HNSC, KIRC, LUAD, LUSC and BRCA, higher VM score were related to poorer survival. On the contrary, in COAD, SKCM, ESCA, UCEC and PRAD, higher VM score scores were associated with better survival (Figure 3A). These results suggest that VM is detrimental to prognosis in most cancer types, which is consistent with previous reports. We further explored its survival effects in a pan-cancer context. We first divided the patients into four groups according to the quartile of VM score. The Kaplan-Meier survival analysis plot indicates that the survival rates of different quartile groups vary that the group with the higher score has worse survival. In particular, patients in the highest quartile group have a significantly worse prognosis compared to other groups (Figure 3B). For the convenience of follow-up research, we divided the tumor samples into two groups according to the median of VM score. The Kaplan-Meier survival plot shows that patients in the low group had a significantly better overall prognosis than patients in the high group (Supplementary Figure S3). Since VM is a novel type of tumor microcirculation model, which is commonly found in highly invasive malignant tumor cells, we further compared the distribution of VM score among tumors of different clinical stages (Figure 3C). As expected, VM score increases gradually with the increase of tumor stage. In addition, higher VM score were also seen in metastatic tumors (Figure 3D). The above results indicate that VM is a biological marker of poor prognosis and may promote tumor progression and metastasis.

### Vasculogenic mimicry is associated somatic mutation at pan-cancer

**FIGURE 4:**
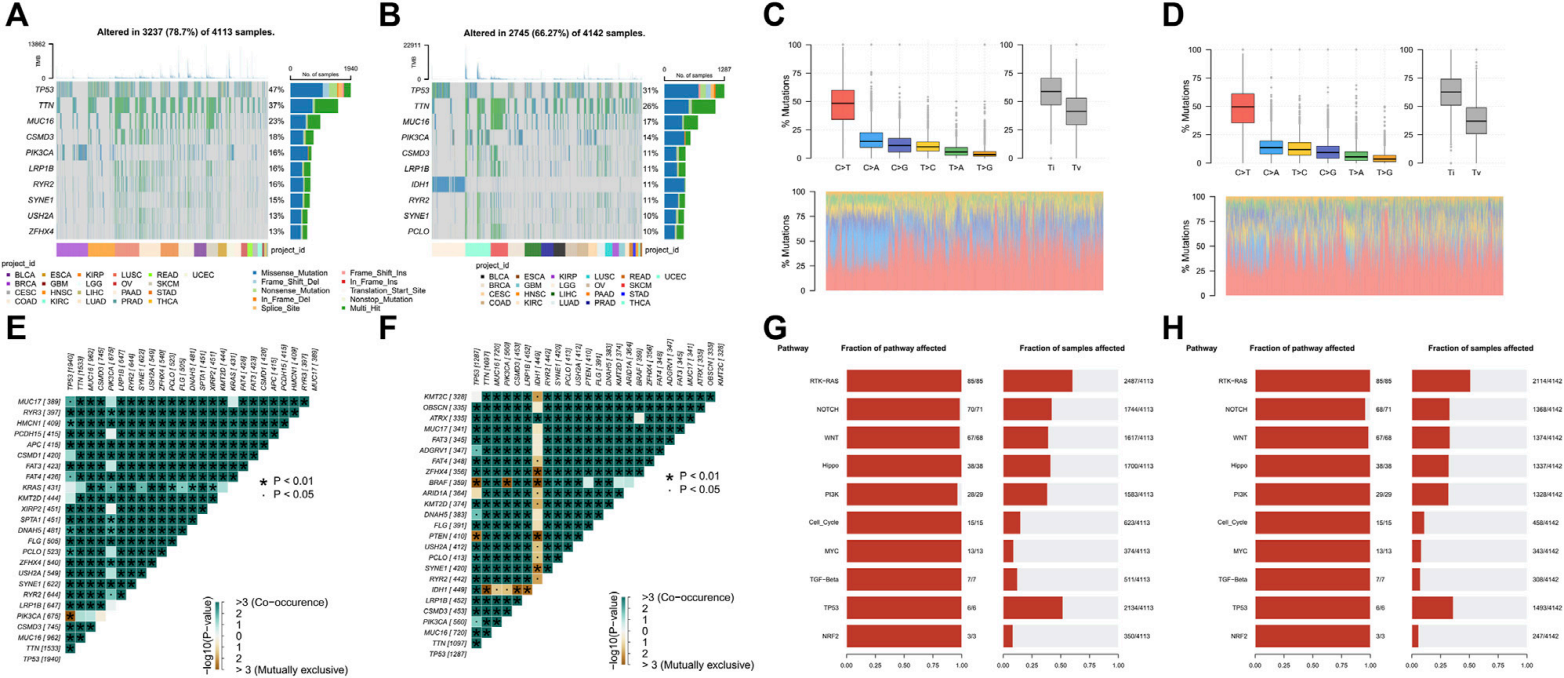
Vasculogenic mimicry (VM) is associated somatic mutation at pan-cancer **(A, B)** The top 10 genes with the highest mutation frequency of high **(A)** and low VM score groups **(B)** and the distribution of different mutation types in pan-cancer. **(C, D)** The proportion of transitions and transversions, and the overall distribution of the six different substitution of a single base in high **(C)** and low VM score groups **(D)**. **(E, F)** Somatic interactivity mutations are co-occurring in the high VM score group **(E)**, while IDH has extensive exclusiveness in the low VM score group **(F)**. **(G, H)** Oncogenic pathways mutations in high VM score group **(G)** and low VM score group **(H)**.

Genomic instability is a hallmark of cancer and is characterized by a high frequency of mutations and chromosomal rearrangements, which is thought to be a driving force behind the oncogenic transformation of tumor evolution (Gorodetska et al., 2019). Since VM was validated to be associated with tumor progression, we further explore the differences in somatic mutation landscape between high VM score group and low VM score group at pan-caner level. We firstly identified the top mutated genes in two groups, respectively. TP53, TTN, MUC16, CSMD3, and PIK3CA were the top five mutated genes in two groups, with significantly higher mutation frequency in the high VM score group than that in the low VM score group. In addition, ZFHX4, USH2A have a higher mutation frequency in the high VM score group, while IDH1, PCLO ranks higher in the mutation frequency ranking in the low VM score group (Figures 4A,B). Furthermore, High VM score group also had higher variants per sample. And among the gene mutation types, most of them are missense mutations (Supplementary Figures S4A, B). Allele frequency analysis showed that the TP53 allele frequency in tumor samples in the high VM score group was less than 50% while more than 50% in the low VM score group (Supplementary Figures S4C, D). To further explore the detailed mechanisms behind the various landscapes of somatic mutations, SNP-based analysis showed that C>T was the most dominant mutation form between the two groups, and the frequency was higher in the low VM score group. And the frequency of transitions in the high VM score group is lower than the frequency of transitions in the low VM score group (Figures 4C,D). Mutual exclusion and co-occurrence analysis showed that there was a significant mutual exclusion of mutations in TP53/PIK3CA in the high VM score group (Figure 4E). There are many mutually exclusive mutations in the low group, among which IDH1 is mutually exclusive with other genes except TP53 and ATRX (Figure 4F). To understand the potential impact of these different somatic mutational backgrounds on the biological behavior of tumors. Analysis of the number and proportion of gene mutations in 10 oncogenic signaling pathways showed that although most pathways had a high proportion of mutations between two groups, the frequency of gene mutations was higher in the high VM score group. Moreover, the proportion of NOTCH signaling pathway affected in the high VM score group is also higher, indicating that NOTCH signaling may potentially regulate VM (Figures 4G,H). The above results show that samples with high VM levels have more mutations, and the oncogenic pathways are greatly affected by mutations, which further indicates that their malignancy is higher.

### Vasculogenic mimicry is associated with immunosuppressive microenvironment

**FIGURE 5:**
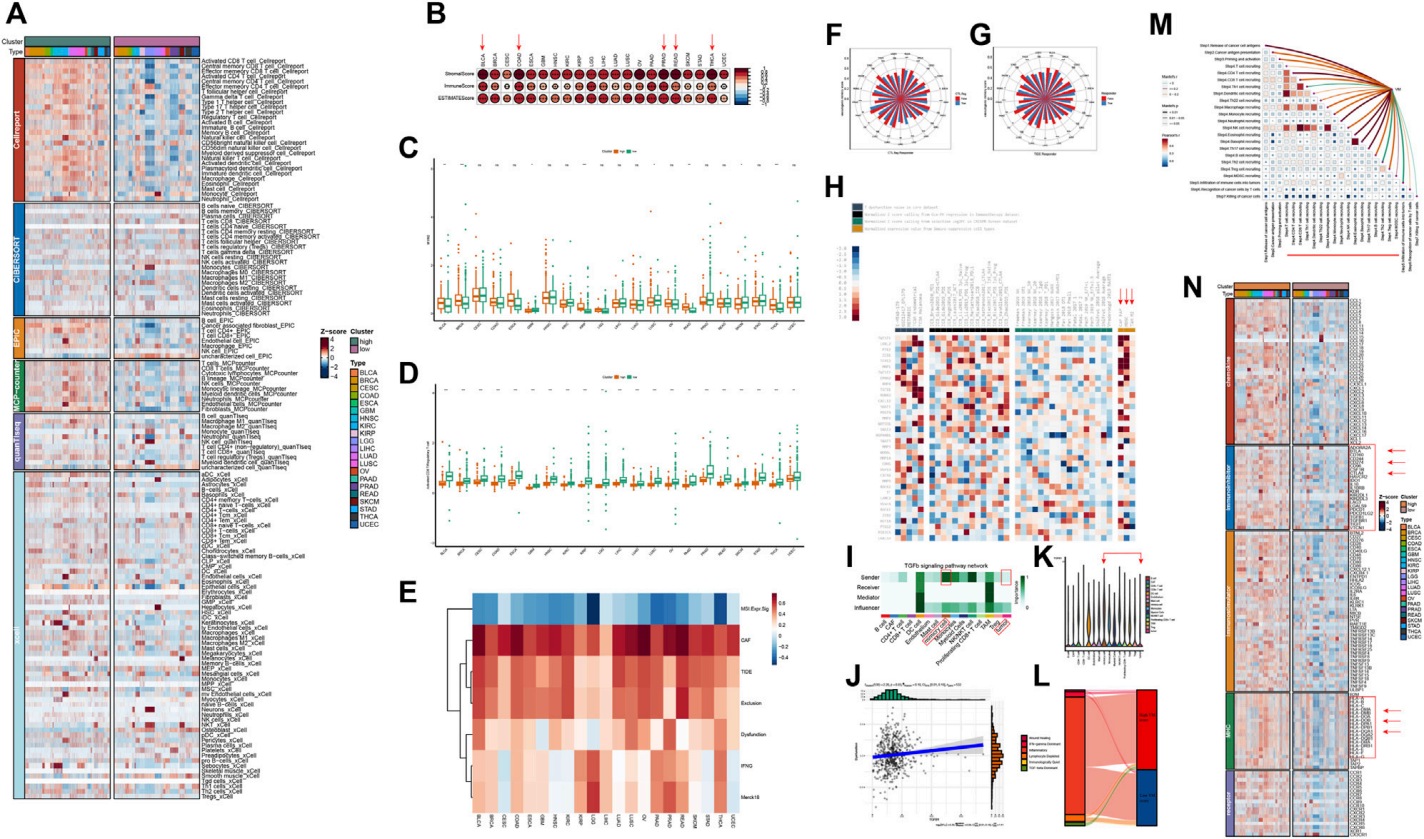
Vasculogenic mimicry (VM) is associated with immunosuppressive microenvironment **(A)** Heatmap shows the infiltration levels of different cellular components in the tumor microenvironment (TME) of different VM score groups. The results are based on six different TME algorithms. **(B)** VM score is positively associated with immune score, stromal score and ESTIMATE score calculated by ESTIMATE algorithm in different tumor types. **(C)** Differences in M1/M2 ratio between high and low VM score groups in different tumor types. **(D)** Differences in activated CD8^+^ T/Regulatory T cell ratio between high and low VM score groups in different tumor types. **(E)** Correlation between VM score and TIDE results. **(F)** The rose chart shows that VM score is lower in the samples with CTL. flag is true. **(G)** The rose chart shows the immunotherapy prediction results of TIDE. Patients with immune response have lower VM score. **(H)** Regulator prioritization results of TIDE. **(I)** Cellchat identifies dominant senders, receivers, mediators and influencers in the TGFb1 intercellular communication network. **(J)** The relationship between TGFβ1 and immune cell dysfunction in KIRC. **(K)** TGFβ1 expression levels in different cell types in KIRC TME. **(L)** Sankey diagram shows the distribution of different immune subtypes. **(M)** Correlation between VM and immune response steps. The heatmap shows the correlation between each immune step. The line between the VM and each step represents the correlation. Red represents positive correlation, and green represents negative correlation. **(N)** Immunity-related factors, including chemokines, MHC molecules, immunostimulators, and immunoinhibitors were highly expressed in high VM score group.

Immune cells infiltrating in the TME interact with tumor cells and jointly affect tumor development and metastasis. Therefore, we further analyzed the relationship between VM and tumor immunity and tumor microenvironment. We selected six algorithms including Cellreport, CIBERSORT, EPIC, MCP-counter, quanTIseq and xCell to quantify the diverse types of cells within TME of different tumors. Strikingly, as shown in Figure 5A, the results of six different algorithms consistently show that patients with higher VM score have significantly higher infiltration level of immune cells. Moreover, the content of CAFs in the high VM score group was also significantly higher than that in the low VM score group. To verify these findings, the immune score, stromal score and ESTIMATE score were also found to be significantly positively correlated with VM levels in all tumors, especially BLCA, COAD, PRAD, READ and THCA (Figure 5B). These above results indicate that the high VM score group has higher levels of immune infiltration, more stromal cells and more complex components in its microenvironment, which may have more complex cell interaction patterns. Considering that regulatory T cells and M2 are important immunosuppressive cells in the TME and are widely used to evaluate the level of immunosuppression (Luckheeram et al., 2012; Ju et al., 2021). While CD8^+^ T cells and M1 are considered anti-tumor immune cells. We therefore examined the ratio of between CD8^+^ T cells and regulatory T cell, M1 and M2 to evaluate immunological status in different groups. We found that in BLCA, BRCA, KIRP, THCA, and PAAD, the ratio of M1 to M2 was higher in the high VM score group. In GBM, LUSC, and SKCM tumors, the ratio of M1 to M2 in the low VM score group was higher. In most tumors, there was no statistical difference between these two groups (Figure 5C). Different from the macrophage ratio results, based on the prediction results of cellreport, we found that the ratio of activated CD8^+^ T cells to regulatory T cells in the low VM score group was significantly higher than that in the high VM score group in all tumors (Figure 5D). The above results suggest that although the level of immune infiltration is higher in high VM score group, its infiltrating immune cells may be in an inactivated or suppressed state. This further indicated that the high VM score group was in a more malignant state. Except for the abundance of immune cell in TME, we also evaluate the functional status of these cells, which showed that the VM score was positively correlated with the immune cell dysfunction, exclusion and TIDE score of the tumor, which illustrates that the high VM score group is immunosuppressed (Figure 5E). It is noteworthy that a high correlation between VM score and CAFs was also observed, indicating that CAFs may play an important role in VM (Figure 5E). We also found that VM score is lower in samples with CTL. flag is true (Figure 5F). And the results of further immunotherapy prediction also showed that the low VM score group may have a better treatment response, which is consistent with the result that VM level is negatively correlated with MSI (Figure 5G). According to the existing studies, MSI-H tumors tend to have a better immunotherapy response (O'Malley et al., 2022). The gene raking results analyzed by TIDE regulator prioritization module showed that most genes were negatively correlated with immunosuppression. Interestingly, some genes are strongly correlated with CAFs and Myeloid-derived suppressor cells (MDSC), which were recognized to play an important role in immune suppression (Figure 5H). Therefore, we hypothesized that VM is involved in immunosuppression in the microenvironment. Analysis of cell communication at the single-cell level based on KIRC provides us with some evidence. Compared with malignant cells, VM cells emit stronger TGFb1 pathway signals, which may promote the TAM-mediated immune suppression process (Figure 5I). Further analysis also showed that TGB1 is positively related to immune dysfunction and VM cells are also the type of cell with the highest TGB1 expression level in the microenvironment of KIRC patients (Figures 5J,K). Previous study has defined the tumor immune microenvironment into six subtypes, including wound healing, IFN-γ dominant, inflammatory, lymphocyte depleted, immunologically quiescent and TGF-β dominant (Thorsson et al., 2018). Analysis at the pan-cancer level found that TGF-dominant subtype was found to have the highest VM score (Supplementary Figure S5A). Moreover, KIRC patients with TGF-beta Dominant subtype were considered to belong to high VM score group (Figure 5L). Furthermore, we also found that VM cells, who are mainly influencer and mediator, have extensive and unique interactions with immune cells in the microenvironment through the LAMININ pathway (Supplementary Figures S5B–D). There have been previous reports that laminin is involved in immunosuppression, which indicates that the LAMININ pathway may also be an important way for VM to promote or mediate the formation of a suppressed immune microenvironment (Simon et al., 2019; Phillippi et al., 2020; Lepucki et al., 2022; Flies et al., 2023). Moreover, we further analyzed the correlation between VM and immune response steps in KIRC. Similar to the previous results, VM was related to the immune cell infiltration process, but was negatively correlated with the immune effector stage (Figure 5M). Similar phenomena are observed in most other tumors (Supplementary Figure S5E). Multiple previous studies have shown that immune checkpoints play a crucial role in cancer immune evasion (Gordon et al., 2017; Jiang et al., 2019). In addition, increasing evidence also suggested that some MHC molecules, such as HLA-E, can also drive tumor immunosuppression (Salomé et al., 2022; Liu et al., 2023). Thus, we analyzed the expression of immune-related molecules between the two groups. Our results shows that the most of MHC molecules and immunoinhibitors were highly expressed in high VM score group (Figure 5N). The above results indicate that VM is related to the immune microenvironment and may be involved in shaping the immunosuppressive microenvironment.

### Single-cell analysis explains potential regulatory mechanisms of vasculogenic mimicry

**FIGURE 6:**
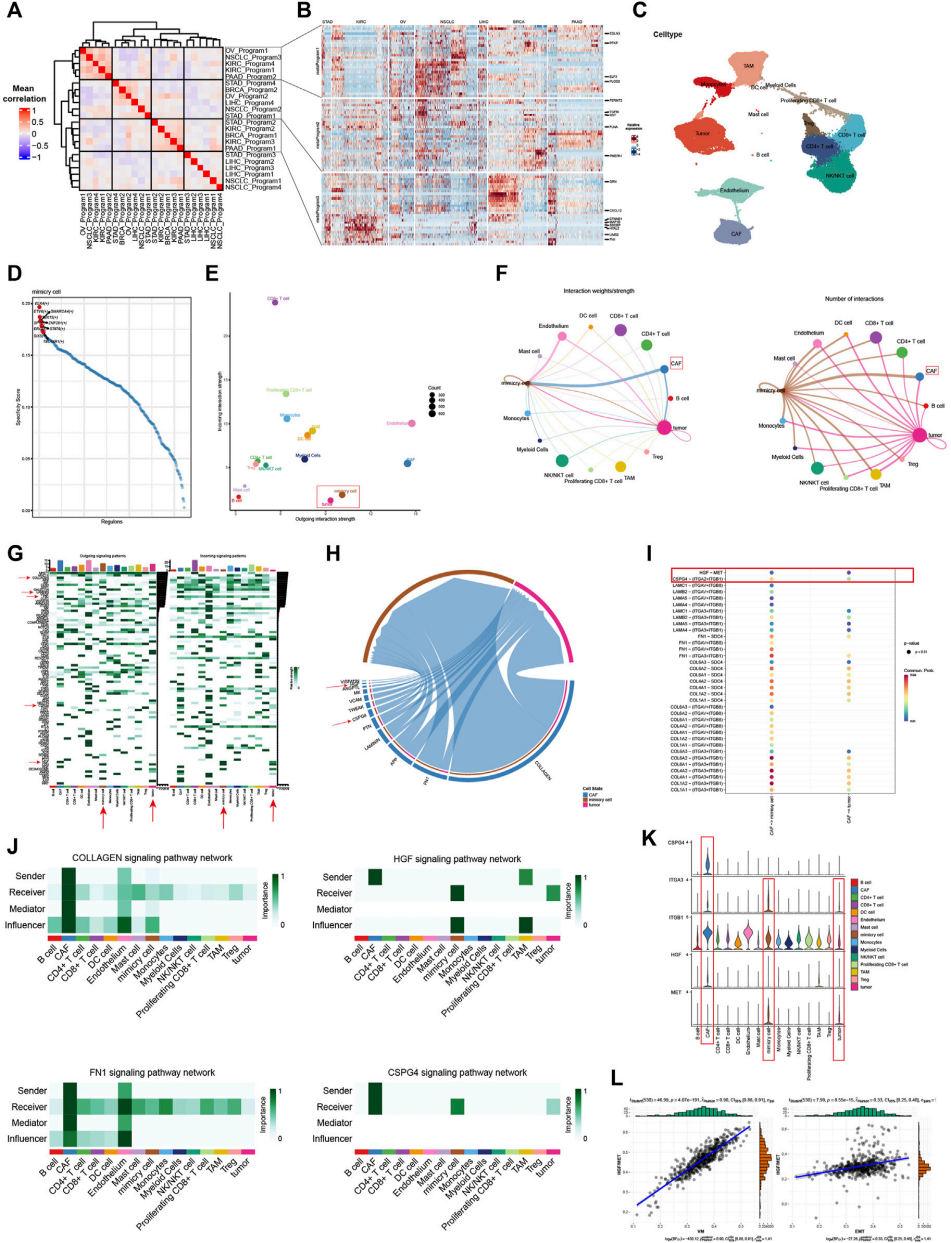
Single-cell analysis explains potential regulatory mechanisms of vasculogenic mimicry **(A)** Correlations between gene modules inferred by NMF. Three highly correlated gene programs were identified. **(B)** The expression of genes in the three gene programs. Red represents high expression, while blue represents low expression. The darker the color, the higher or lower the expression. **(C)** UMAP plot shows various cellular components in the KIRC tumor microenvironment (TME). **(D)** Top 10 transcription factors enriched in VM cells. The higher the specificity score, the greater the association with VM. **(E)** The stength of signals emitted and received by different cellular components in the TME. The larger the value on the *y*-axis, the more signals are received, and the larger the value on the *x*-axis, the more signals are emitted. The size of the point represents the amounts of cells. **(F)** Cell communication circle diagram. Demonstrating the communication landscape of tumor and VM cells when they serve as senders and receivers. **(G)** Demonstrate cell communication pathways in the TME and the contribution of different cells to different signaling pathways. The darker the color, the greater the contribution. **(H)** Chord diagram shows the interaction pathways between CAFs, VM and tumor cells. The wider the channel, the higher the strength. **(I)** Communication possibility based on ligand receptor pairs. Red represents a higher possibility of communication. **(J)** Cellchat identifies dominant senders, receivers, mediators and influencers in Collagen, HGF, FN1 and CPSG4 signaling intercellular communication network. **(K)** The violin plot shows the expression of HGF and CSPG4 pathway ligand receptor pairs in different cells. **(L)** Positive correlation was observed between HGF/MET score and VM, EMT in KIRC.

In order to further explore the potential regulatory mechanism of VM and its interaction with the TME, we took advantage of the ability of single-cell data to characterize cell characteristics at the subpopulation level for subsequent analysis. After extracting the malignant cells from seven individual cancer types, we calculated the VM score for each tumor cells and considered cells in the top 10 percent of the scores in each cancer types as VM cells. Next, NMF was used to identify expression patterns shared across different tumors to explore the underlying mechanism of VM. As shown in Figure 6A, three expression programs that including highly correlated were identified by using gene module correlation analysis. We focused on the top 30 genes in these expression programs to explain the biological functions. The genes of metaprogram one are enriched in glycolysis, which are considered to be metabolism-related modules. Metaprogram two contains EMT genes, which are also highly expressed in VM cells, are considered to be EMT modules. Metaprogam3 mainly contains genes related to cell growth and migration. The above results further strengthen the evidence of the connection between EMT and VM, and also suggest that the biological process of VM may be the product of a hypoxic microenvironment because its glycolysis metabolic process. These results also suggest that our analysis using the gene set score is reasonable.

Next, scRNA-seq data from 19 different renal carcinoma samples was integrated with batch effects eliminated by using RunHarmony function (Supplementary Figure S6A). Based on the graph-based clustering analysis, resolution is set equal to 0.3 and cells were divided into 18 cell clusters and were annotated to seven cell types using known lineage marker (Supplementary Figures S6B, C). In order to identify potential regulators of the biological process of VM, we performed single-cell regulatory network inference and clustering (SCENIC) analysis and illustrated the top 10 most active transcription factors in VM cells (Figure 6D). Among them, some transcription factors have been reported to mediate the EMT process, such as TBL1XR1, STAT6, ETV6, and SMARCA4, indicating their potential to regulate VM and promote tumor invasion (Fararjeh and Liu, 2019; Zhao et al., 2019; Chen et al., 2023; Xu et al., 2023). Furthermore, SP1 has also been reported to mediate VM formation by interacting with the TWIST/VE-cadherin/AKT pathway (Han and Lee, 2022).

We hypothesized that different cell populations in the microenvironment participate in complex crosstalk and interaction patterns with VM cells. Thus, CellChat was used to analyze and visualize the communication strength and number of each cell in the tumor environment to further analyze the at the single-cell dimension (Figures 6E,F). VM cells have extensive communication with various components in the microenvironment, and their communication intensity and number are much higher than that of tumor cells, indicating that they are active in the microenvironment (Figure 6F). In addition, we were surprised to find that the number of interactions between CAFs and VM cells was much greater that of cancer cells (Figure 6F). Our previous results also found that there is a strong correlation between VM score and the infiltration level of CAFs, which indicates that CAFs may be involved in plays an important role in VM. Further analysis also found that the active signaling pathways of VM cells are significantly different from those of malignant cells, and that VM cells have unique communication patterns in the microenvironment (Figure 6G). Therefore, we further explored the interaction patterns and signaling pathways between CAFs and VM, malignant cells. We found that communication is mainly active in multiple signaling pathways that were known to regulate EMT and VM, such as Laminin signaling, Collagen signaling, HGF signaling, FN1 signaling and CPSG4 signaling (Figure 6H). We also show the crosstalking ligand-receptor pairs of signaling pathways that are significantly upregulated in VM cells (Figure 6I). The results show that strong communication between CAFs and VM cells occurs mainly between ligands such as collagen (types I, IV and VI), fibronectin (FN1), laminin (LAMB2, LAMA4, LAMA5, LAMC1), CSPG4 and HGF and receptors such as integrins family (ITGA2, ITGA3, ITGAV, ITGB8, ITGB1), SDC4 and MET (Figure 6I). We further show the role and signal intensity of the microenvironment in the above-mentioned signaling pathways. It can be seen that CAFs is the main sender of the Collagen and FN1 signaling pathways, and VM cells also participate in communication as important influencers (Figure 6J). It indicated that CAFs may mediate VM through these pathways. It is worth noting that HGF and CSPG4 are specific communication modes for VM cells and malignant cells. And HGF is jointly emitted by CAFs and affected by TAMs. CSPG4 is only released by CAFs and acts on VM and malignant cells (**Figure 6K**). It has been previously reported that HGF/MET is associated with EMT and promotes the generation of VM(21). This indicates that HGF and CSPG4 are likely to be the potential mechanisms by which CAF regulates VM. We further demonstrated the expression of ligand receptor pairs of the above two pathways. It can be seen that CSPG4 and HGF are mainly expressed in CAFs, and the expression of the corresponding receptors in mimic cells is also significantly higher than that in malignant cells (Figure 6K). Bulk-based data analysis also showed that HGF/MET ligand receptor pairs were positively correlated with VM score and EMT (Figure 6L). The above results emphasize the relationship between EMT and VM, and further point out the potential regulatory mechanism by which CAFs promotes VM.

### Vasculogenic mimicry is associated with drug sensitivity and immunotherapy outcome

**FIGURE 7:**
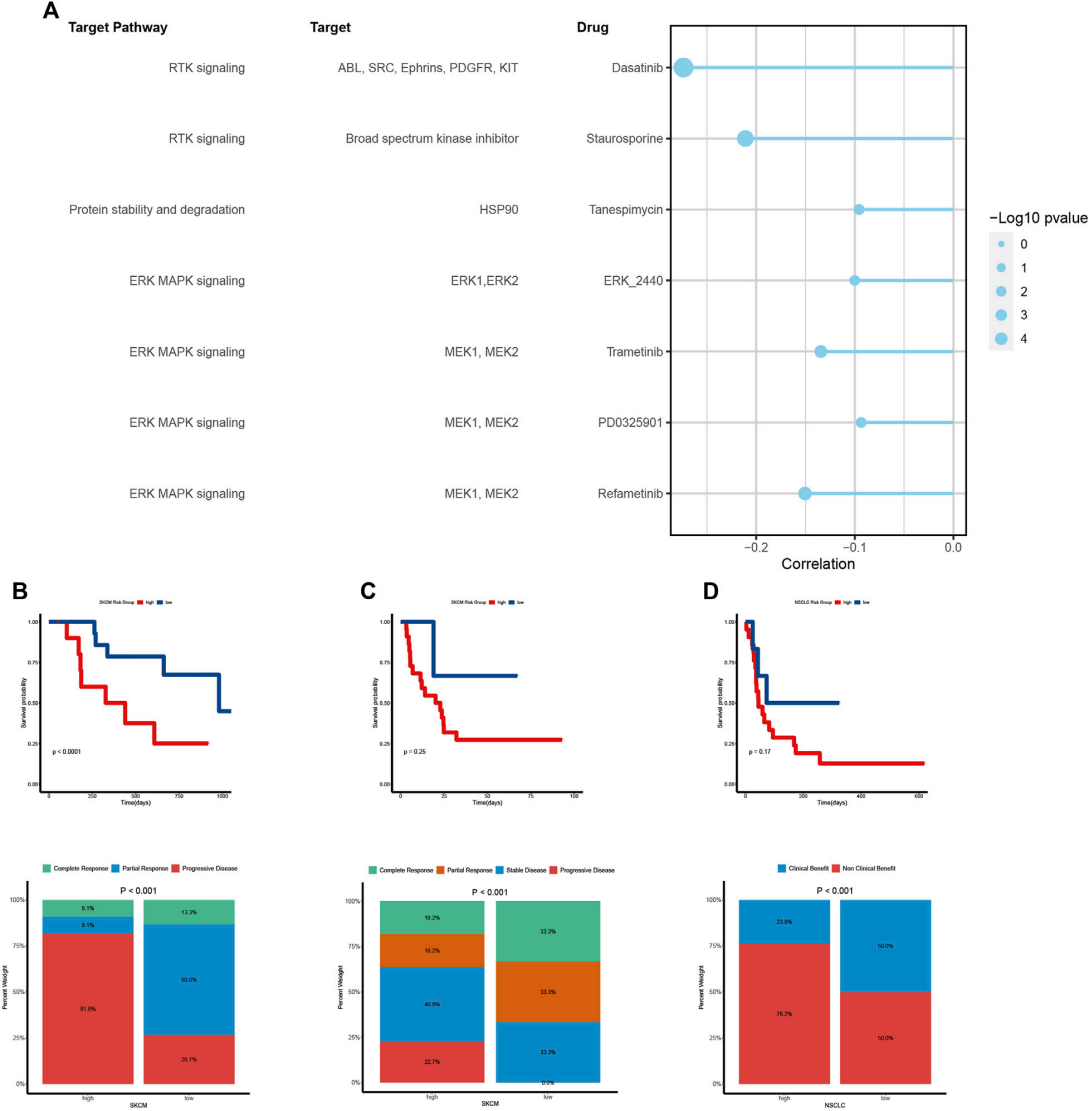
Vasculogenic mimicry (VM) is associated with drug sensitivity and immunotherapy outcome **(A)** The relationship between VM score and drug sensitivity (IC50 value). Each row represents a drug and drug target. The length of the line represents the correlation coefficient. The blue represents a negative correlation. The size of the point represents the statistical significance. **(B, C, D)** Kaplan–Meier curves show the difference in survival prognosis between high and low VM score groups in two SKCM cohorts **(B, C)** and a NSCLC cohort **(D)**. The stacked column chart shows the immune response. Higher response levels were investigated in low VM score group.

Previous results in this paper have shown that VM is closely related to tumor prognosis, invasion, metastasis, microenvironment and immune status. At present, traditional anti-angiogenic drugs have no obvious effect on inhibiting the formation of VM in malignant tumors (Angara et al., 2017; Fathi Maroufi et al., 2020; Luo et al., 2020). Thus, we further investigate to explore potential therapeutic drugs for VM. Through calculating the correlation between VM score and half-maximal inhibitory concentration (IC50) of drug treatment response in GDSC, we screened out seven drugs that were negatively correlated with IC50 as potential therapeutic agents (Figure 7A). These seven drugs mainly targeted ERK/MAPK and RTK pathways. The ERK/MAPK pathway is an important signal transduction pathway that plays a key role in cellular processes such as growth, proliferation, differentiation, and survival, which can promote VM by activating EMT(5,21,61,84,85). In view of the fact that previous results have found that VM is closely related to the tumor immune microenvironment and immune status, and the prediction results of TIDE also showed that the VM score has the ability to predict the effect of immunotherapy. We used the immunotherapy cohort to test the predictive ability of the scoring system. Consistent with the previous results, the low VM score group had a better response to immunotherapy and could obtain a better immunotherapy response. However, further analysis of clinical prognostic data found that there was no statistical significance in survival between the two groups, indicating that the prognosis may be affected by other factors, and more detailed grouping of VM is needed to achieve precise treatment to benefit patients (Figures 7B–D).

### VRS can predict prognosis and immunotherapy outcomes in KIRC patients

**FIGURE 8:**
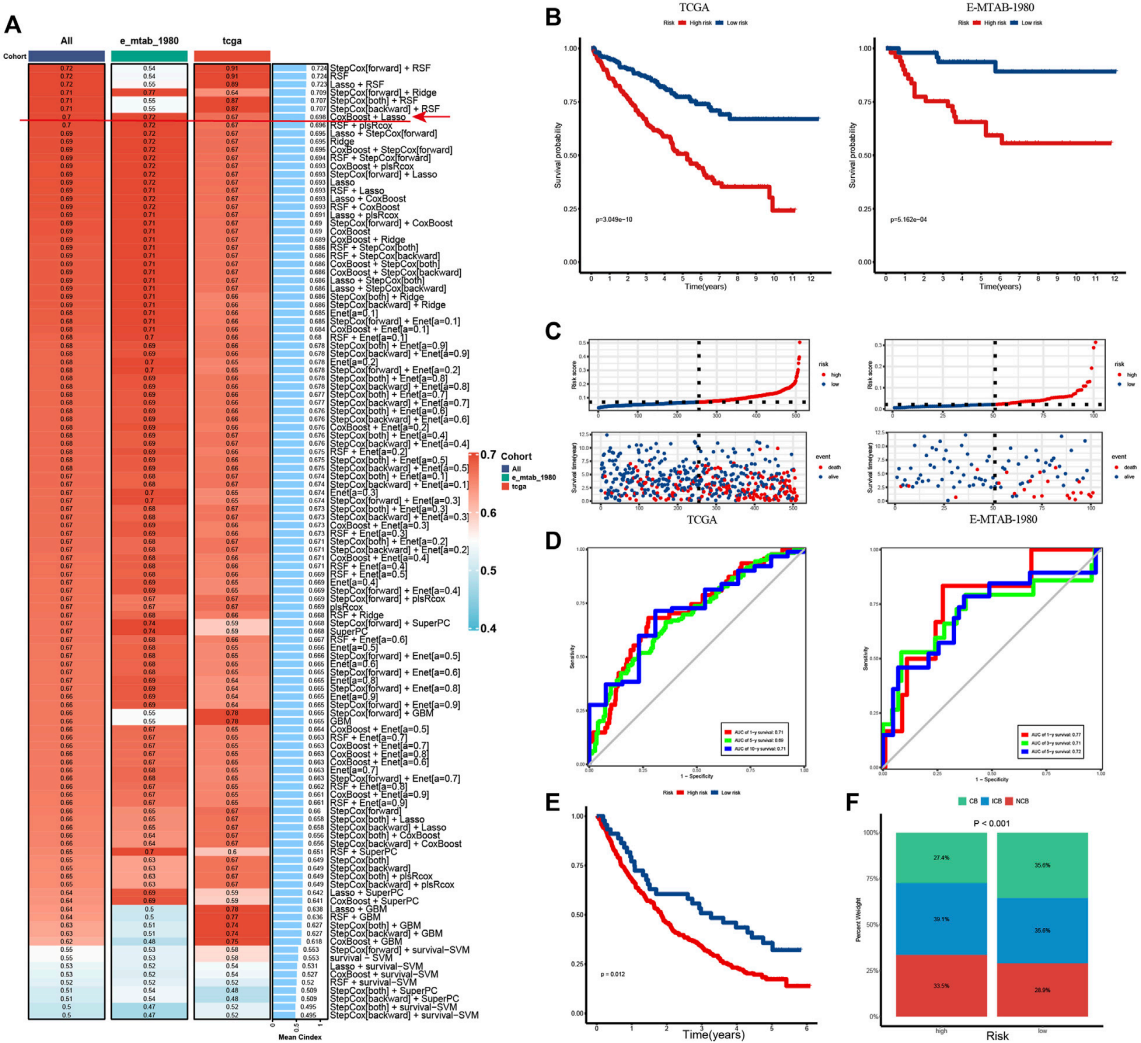
VRS can predict prognosis and immunotherapy outcomes in KIRC patient **(A)** C-index for 101 machine learning algorithm combinations in training and validation cohorts. **(B)** Kaplan–Meier curves show survival differences between high and low risk groups in the TCGA (left) and E-MTAB-1980 cohort (Right). **(C)** Distribution of survival status and survival time of patients in high and low risk groups in the TCGA (left) and E-MTAB-1980 cohort (Right). **(D)** Time-dependent ROC curves of the model in the TCGA (left) and E-MTAB-1980 cohort (Right). **(E)** Kaplan–Meier curve show survival differences between patients in high- and low-risk groups in immunotherapy cohort. **(F)** Stacked column chart showing the proportion of patients benefiting from immunity within different risk groups. ROC, receiver operating characteristic; AUC, area under the ROC curve. CB, clinical benefit; ICB, intermediate clinical benefit; NCB, no clinical benefit.

To promote clinical translation, we developed a VRS system in KIRC. First, we identified 20 potential prognosis-related VM genes and incorporated them as potential prognosis biomarkers into the integrated machine learning program (Supplementary Figure S7A). The CoxBoost + Lasso achieved a high C-index on both training and validation data sets, making it the best prognosis model (Figure 8A). The method selected 11 genes, and their coefficients are shown in Supplementary Figure S7B. Based on median of VRS, we divided patients into high and low risk groups. As expected, patients with higher risk had worse prognosis than those with lower risk, and patients with higher risk scores had shorter survival periods and lower survival rates (Figures 8B,C). The 1-, 3- and 5-year AUC of TCGA cohort were 0.71, 0.69 and 0.71 respectively, and E-MTAB-1980 cohort were 0.77, 0.71 and 0.72 respectively. The above results prove the robustness of the model (Figure 8D). Moreover, this model was also proven to be an independent prognostic factor in KIRC patients (Supplementary Figures S7C, D). Next, the model was also shown to have the potential to predict immunotherapy outcomes. Patients with low risk were more likely to benefit from immune treatment, and had higher response rates after treatment (Figures 8E,F).

## Discussion

Aberrant activation of angiogenesis within tumor microenvironment is considered as a hallmark of cancer (Hanahan and Weinberg, 2011). While tumor cells can exchange nutrients and metabolic products with their surrounding environment via simple diffusion, the formation of blood vessels becomes essential when the tumor diameter surpasses 2 mm to ensure an adequate supply of nutrition and oxygen (Folkman, 1971). Tumor-induced angiogenesis play a pivotal role in rapid growth, infiltration, and metastasis of malignant cells to other organs (Weidner et al., 1991). VM is a unique vascular structure distinct from the classical tumor angiogenesis pathway and formed by tumor cells themselves mimicking endothelial cells to provide blood perfusion (Maniotis et al., 1999; Hendrix et al., 2003). However, a comprehensive analysis of the role of VM across different cancers is currently lacking. Hence, our research employed a multi-omics approach based on VRGs collected from literature retrieval combined with transcriptomics, epigenetics, genomics, single-cell omics to characterize the potential regulatory mechanisms of VM and further characterized its complex relationships and interactions with the TME. Finally, seven drugs were screened that may benefit tumor patients with VM.

Genes associated with VM contribute to several biological processes, including EMT, CSC, Extracellular Matrix (ECM) remodeling, and hypoxia response (Wei et al., 2021). Our study finds most VRGs are dysregulated. Importantly, genes from the MMP family are significantly upregulated across various tumors. MMPs, a family of zinc-binding metalloproteinases, are involved in the degradation of ECM components and were reported play a pivotal role in the formation of vasculogenic networks by cancer cells (Gorodetska et al., 2019; Benjakul et al., 2022). The expression of genes is commonly regulated by genomics and epigenetics. Analysis of genetic variation indicates a positive association between the copy number of most genes and their gene expression, particularly PTK2 and PIK3CA, which exhibit a high degree of amplification in nearly all tumors. Both PI3K and PTK2 are involved in pathways integral to tumor initiation and progression (Lee et al., 2015; Alqahtani et al., 2019). Previous studies have reported that mutations in PI3K can augment the formation and functionality of VM (Huang et al., 2023). Meanwhile, correlation analysis between methylation levels and transcriptome expression levels reveals a negative correlation for most genes. In conclusion, our findings suggest that aberrant genomic and epigenetic variations could lead to dysregulation in the expression of VRGs, thereby triggering VM.

Next, we explored the characteristics of VM and find a strong correlation with hypoxia, angiogenesis and EMT. Numerous reports have highlighted the critical role of these characteristics in the formation and regulation of VM(4,5). Hypoxia is a trigger factor for angiogenesis (McDonald and Baluk, 2002; Zheng et al., 2021). Previous research reported that HIF can enhance the expression of EMT regulatory factors and ECM remodeling enzymes, thereby conferring plasticity and stemness to tumor cells and promoting tumor invasion and metastasis (Comito et al., 2011; M et al., 2017). EMT, a process that imparts mesenchymal and stem cell-like characteristics to tumor cells, plays a central role in VM (Qi et al., 2014; Yang et al., 2015; Wang et al., 2019; Luo et al., 2020; Kang et al., 2021; Wei et al., 2021; Benjakul et al., 2022). It enables epithelial cells to adopt mesenchymal cell characteristics, thereby facilitating the formation of new vascular structures. Research indicates that EMT can promote the formation of vascular structures by prompting cells to express VRGs. Additionally, EMT can induce VM by augmenting the migratory and invasive capabilities of tumor cells, as well as ECM components and hardness (Luo et al., 2020; Wei et al., 2021). Our findings also demonstrate the highest correlation (R = 0.87) between EMT and VM. Single-cell analysis further reveals EMT as a key feature of VM cells. Furthermore, we discovered that NOTCH and WNT signaling pathways, which are associated with EMT, also exhibit a high correlation with VM. In summary, our results suggest that multiple factors participate in VM, with EMT playing a crucial role in this biological process. It may serve as a core feature and central hub for VM, linking multiple signaling pathways and molecular events to coordinate the induction of VM in tumor microenvironment.

Interactions between TME components and tumor cells constitute a focal point in cancer research (Thorsson et al., 2018; Li et al., 2022; Chen L-X. et al., 2023; Chhabra and Weeraratna, 2023). Our results suggested that samples with a high propensity for VM exhibit elevated levels of immune and stromal infiltration. Earlier studies reported an association between VM and increased immune cell infiltration in cancers such as melanoma, renal carcinoma, and hepatocellular carcinoma (Q et al., 2022; Tan et al., 2022; Zhao et al., 2023). TAMs and CAFs within the TME have also been reported to promote VM formation through interactions with tumor cells (Luo et al., 2020; Barnett et al., 2016; Q et al., 2022; Tan et al., 2022; Pan et al., 2020; Ding et al., 2018; Kim et al., 2019; Liu et al., 2021; Wu et al., 2023). Further analysis reveals that interactions between tumor cells and CAFs and the interactions VM cells and CAFs were the most different, suggesting a potential hub for promoting VM formation. Our results reveal that CAFs interact with VM cells via Laminin, Collagen, and FN1 signaling pathways. Previous research demonstrates that CAFs can modulate the extracellular matrix (ECM) by producing collagen, fibronectin, laminin, and MMPs, thereby creating a conducive microenvironment for EMT in tumor cells, thereby endowing them with VM capabilities (Seftor et al., 2001; Yang et al., 2016; Poltavets et al., 2018). We also found that CAFs interact uniquely with VM cells and tumor cells via HGF/MET and CSPG4 signaling pathways in clear cell renal carcinoma, and its effect on VM cells is significantly higher. HGF is a growth factor known to promote VM signaling pathways and induce EMT across various cancers (Wang et al., 2015; Ding et al., 2018). It can activate multiple signaling pathways such as PI3K/AKT, ERK1/2 signaling, and TWIST1/2 signaling via the HGF/MET signaling pathway (Wang et al., 2015; Han et al., 2016; Ding et al., 2018). This activation also promotes the EMT process and the expression of VM related genes genes. Additionally, prior studies also report that CSPG4 may foster proliferation, migration, and invasion of tumor cells by regulating EMT-related pathways (Winship et al., 2017; Wilms et al., 2022). To sum up, CAFs promote VM formation through multiple pathways including fibronectin and laminin as well as potentially key mechanisms such as HGF/MET and CSPG4 pathways. Our findings underscore the pivotal role of CAFs in VM and reiterate the centrality of EMT in VM.

It is noteworthy that the prognosis of high VM score samples is markedly worse despite higher immune infiltration. Numerous studies have shown that a high content of immune components does not necessarily correlate with a better prognosis, which is influenced by multiple factors such as tumor invasiveness, tumor microenvironment composition, and relative advantage of immune stimulation (Fridman et al., 2017; Li et al., 2019). Previous investigations have indicated that tumor-induced angiogenesis contributes to immune suppression and evasion (Tu et al., 2023). Therefore, we characterized the functional status of immune cells in the TME and revealed a negative correlation between the VM level and the ratio of anti-tumor to pro-tumor immune cells. In addition, we further found that VM is positively correlated with immunosuppressive molecules in the TME. Research have reported that VM TME can induce the expression of immune suppression factors like TGF-β(15). The TGFβ signaling pathway is a critical determinant of immune regulation. TGFβ derived from cancer cells has been proven to promote the transformation of CD4^+^ T cells into Treg cells and exclusion of T cells (Mariathasan et al., 2018; Gu et al., 2022). Our research also indicated that VM cells are a type of cell in TME with the highest expression of TGFβ and interact extensively with immune cells through the TGFβ signaling pathway. In addition, VM cells interact uniquely with immune cells through laminin, which has been reported to have both immune promotion and suppression properties (Huard et al., 1985; Simon et al., 2019; Phillippi et al., 2020; Lepucki et al., 2022; Flies et al., 2023). We speculate that VM cells may mediate immune evasion by utilizing its immunosuppressive effect. Moreover, tumors undergoing VM are often in a severe hypoxic state. Hypoxia and HIF have also been proven to participate in immune evasion (McGettrick and O'Neill, 2020). Notably, high CAFs infiltration in VM TME may also play a significant role in immunosuppression (Koppensteiner et al., 2022). Therefore, we postulate that due to their incomplete morphology, mimicry vessels have higher permeability, facilitating entry of immune cells into tumor tissues. However, when tumor cells form VM, there may be multiple factors in their microenvironment mediating immunosuppression, thereby causing tumor cells evasion from immunity.

Anti-angiogenic therapy has emerged as an efficacious strategy for cancer treatment in recent years, yielding promising results (Huang et al., 2022). However, mounting evidence suggests that tumor samples with VM positive exhibit resistance to prevalent anti-angiogenic drugs such as bevacizumab and sunitinib (Angara et al., 2017; Zhang et al., 2019; Fathi Maroufi et al., 2020; Luo et al., 2020). Some studies even indicate that sunitinib can foster VM and resulting cancer invasion by inducing tumor hypoxia (Zhang et al., 2014; Sun et al., 2017; He et al., 2022). Therefore, developing or finding drugs that can accurately target VM is the focus of research. Utilizing our screening strategy, we identified seven potential drugs targeting VM. These drugs primarily obstruct ERK/MAPK and RTK signaling pathways. Research indicated that both these pathways are involved in the regulation of EMT and VM formation, and targeting them could potentially inhibit or block this biological process (Han et al., 2016; Ding et al., 2018; Luo et al., 2020; Kang et al., 2021; Liu et al., 2022; Du et al., 2023). VM is a prevalent feature of late-stage tumors. The potential clinical application of these drugs in VM positive tumor samples could improve patient prognosis and enable more personalized treatment. Future research should focus on conducting clinical trials to validate these findings.

To summarize, our multi-omics analysis has elucidated the principal regulatory mechanisms and associated factors involved in VM formation. We have also uncovered its potential role in immune suppression. Subsequent analysis has identified potential drugs that target VM. Finally, we constructed a model with good predictive performance in KIRC. Nevertheless, a significant limitation of this study is that our current findings are derived from an extensive analysis of big data. Therefore, a substantial number of experiments are required to validate these results and to uncover potential mechanisms and their clinical implications.

## Conclusion

In summary, we characterized the features of VM based on multi-omics data for the first time at the pan-cancer level. We systematically analyzed the comprehensive alterations in VRGs at the genomic, epigenomic, and transcriptional levels based on the TCGA pan-cancer cohort. Our results support that VM is a manifestation of higher malignancy in tumor patients. Furthermore, our results highlight the critical role of EMT in VM, as well as the important promoting role of CAF in VM formation. Our study elucidates the association between VM levels and immune-suppressive microenvironments across various cancer types, and highlight the intricate interactions between VM and TME. In order to promote clinical translation and precision medicine, a prognostic prediction model and potential targeted drugs have also been identified. Our research offers novel insights into the occurrence of VM and its contribution to tumor progression and drug resistance. A deeper understanding of these relationships could enhance our comprehension of tumor growth and metastasis mechanisms, thereby paving the way for innovative anti-cancer therapeutic strategies.

## Data Availability

The original contributions presented in the study are included in the article/Supplementary Material, further inquiries can be directed to the corresponding author.
