# Supplementary figures and images for: Pan-cancer dissection of vasculogenic mimicry characteristic to provide potential therapeutic targets
Source: Front Pharmacol. 2024 Apr 17;15:1346719. doi: 10.3389/fphar.2024.1346719 (PMC11061449; doi:10.3389/fphar.2024.1346719)

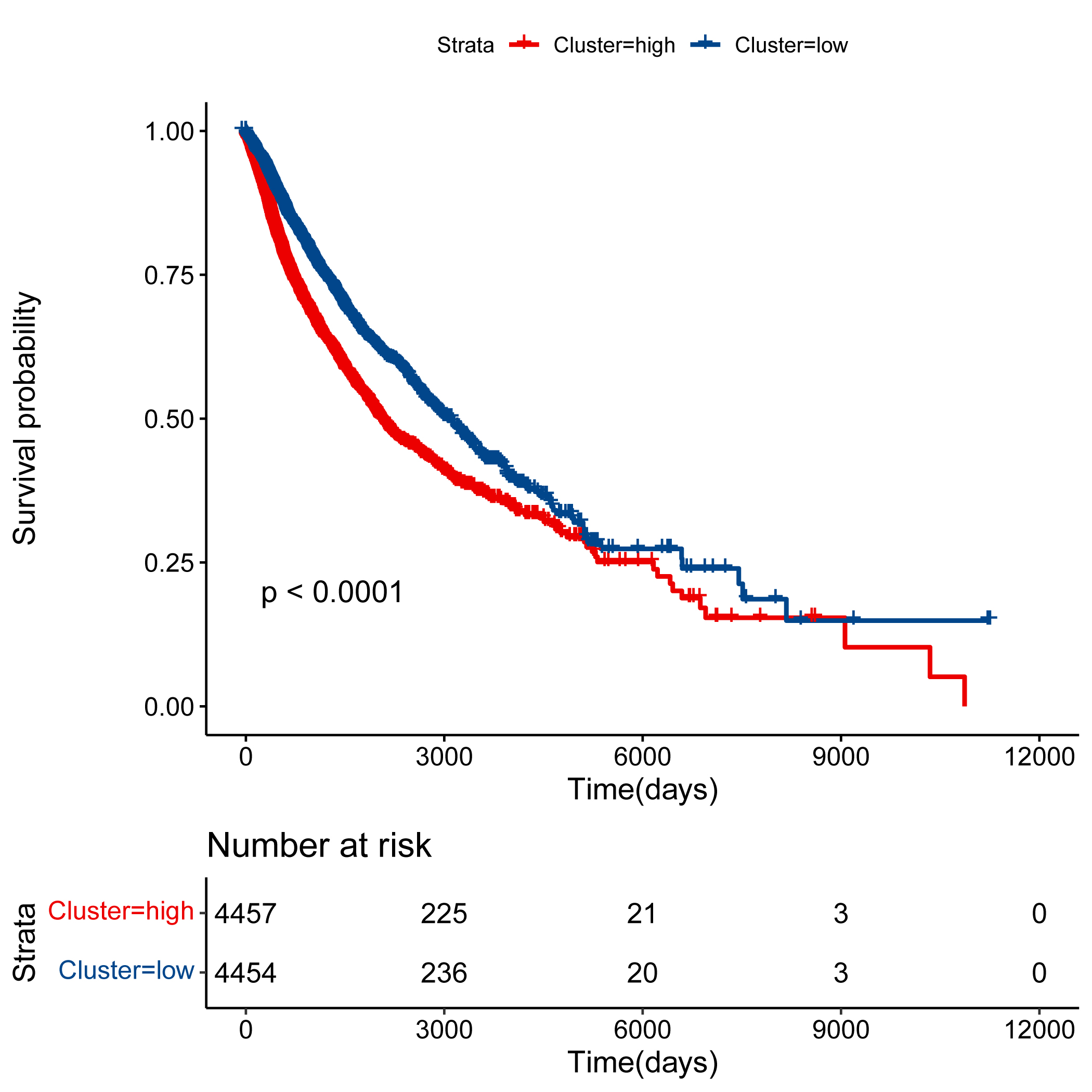

Supplement: Supplementary file 2 [file Image3.JPEG]

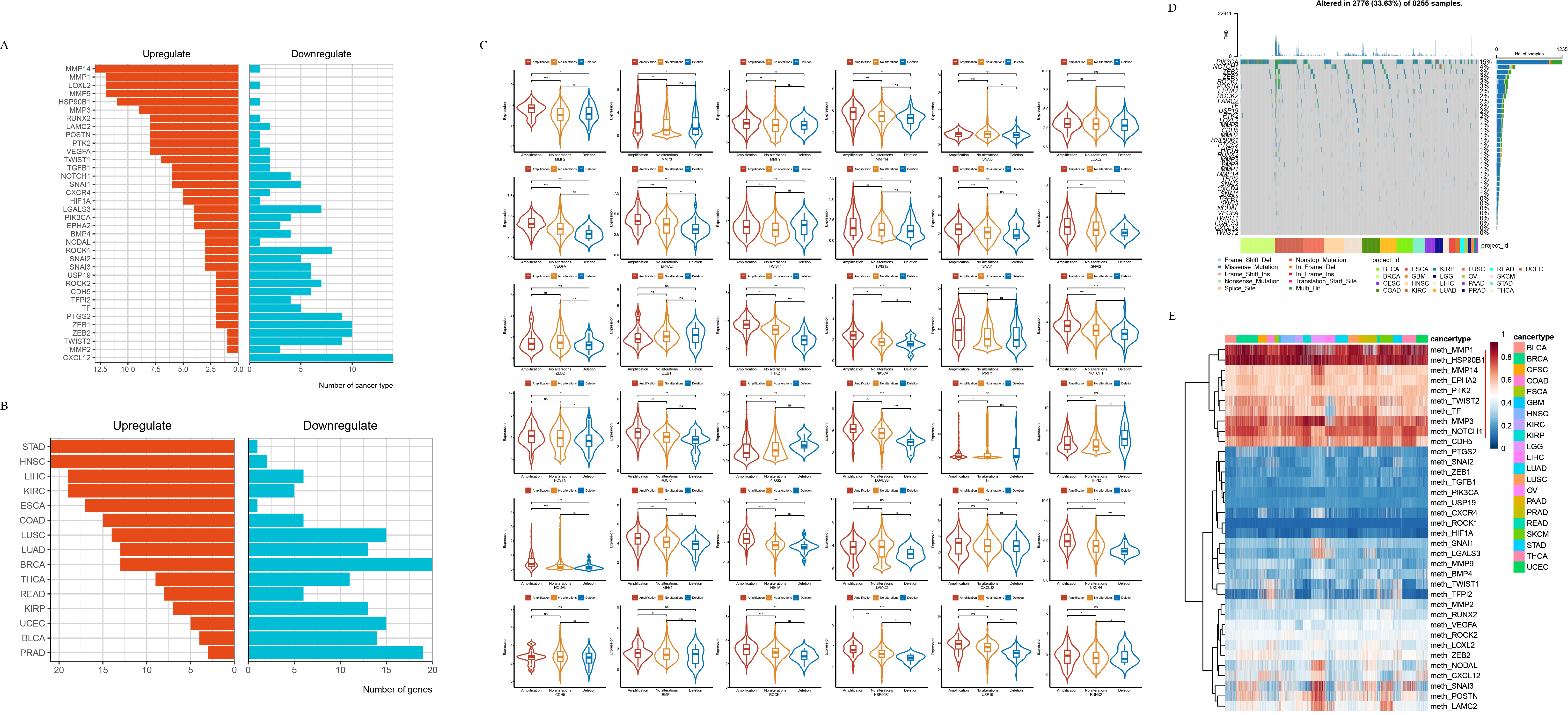

Supplement: Supplementary file 4 [file Image1.JPEG]

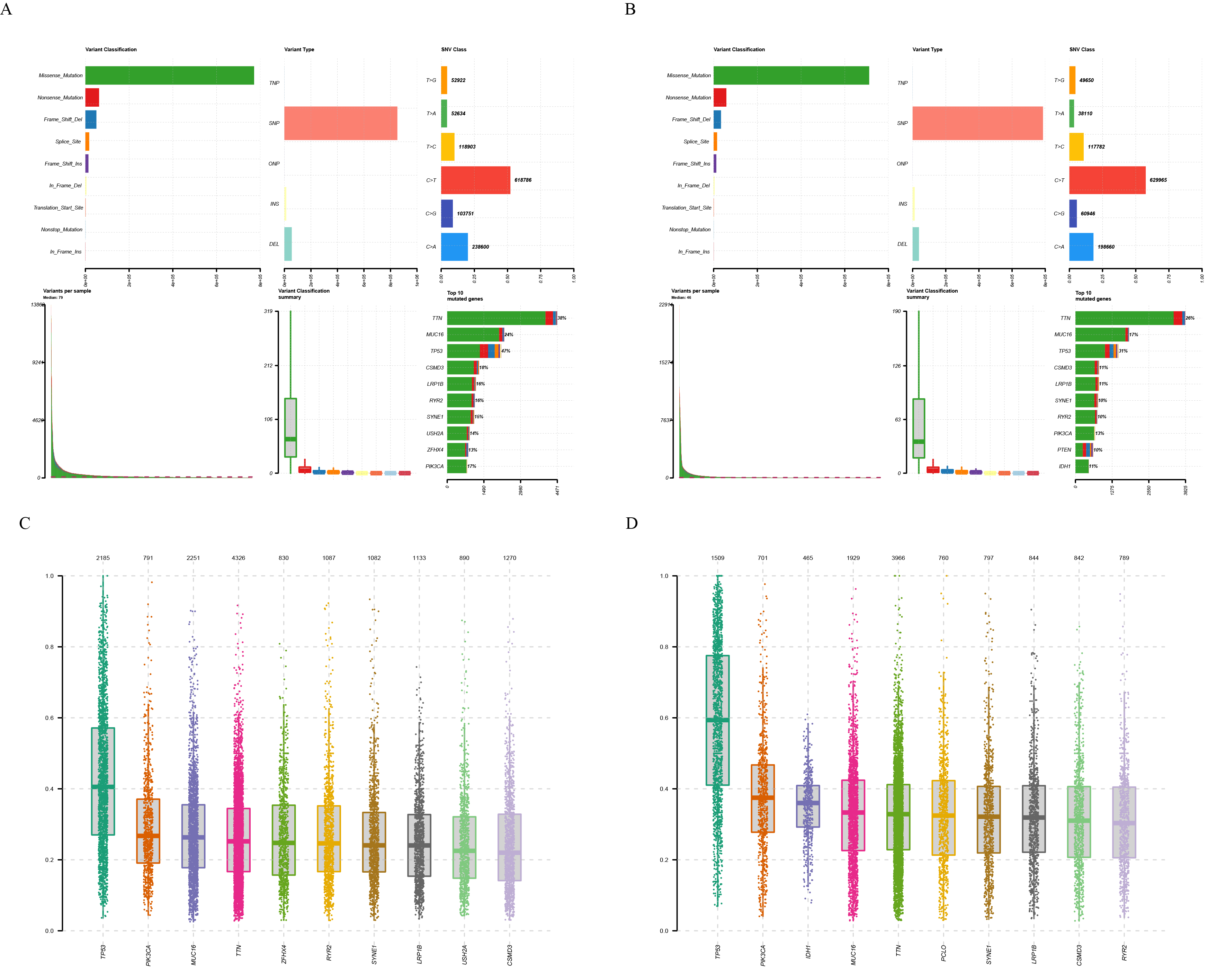

Supplement: Supplementary file 5 [file Image4.JPEG]

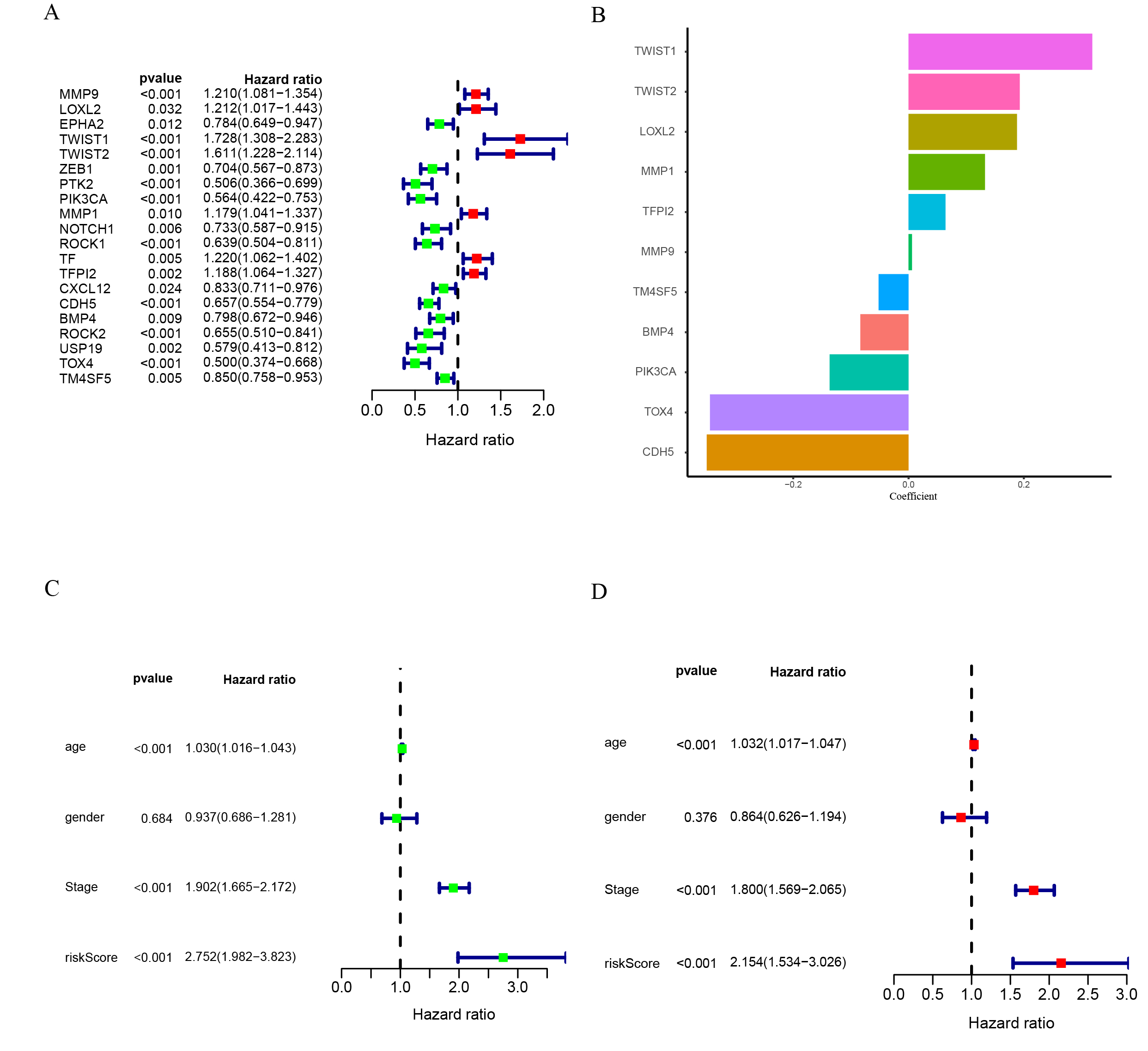

Supplement: Supplementary file 6 [file Image7.JPEG]

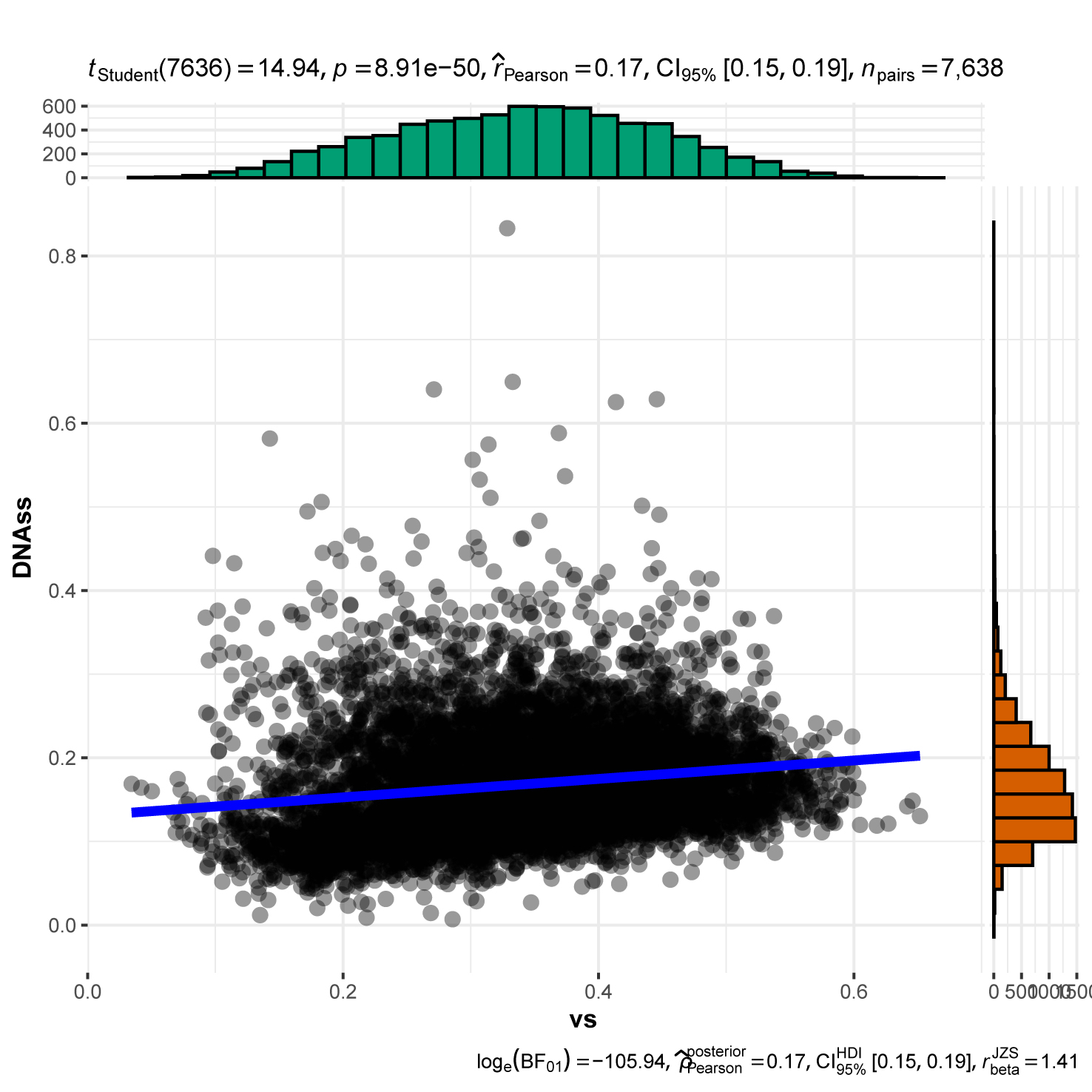

Supplement: Supplementary file 7 [file Image2.JPEG]

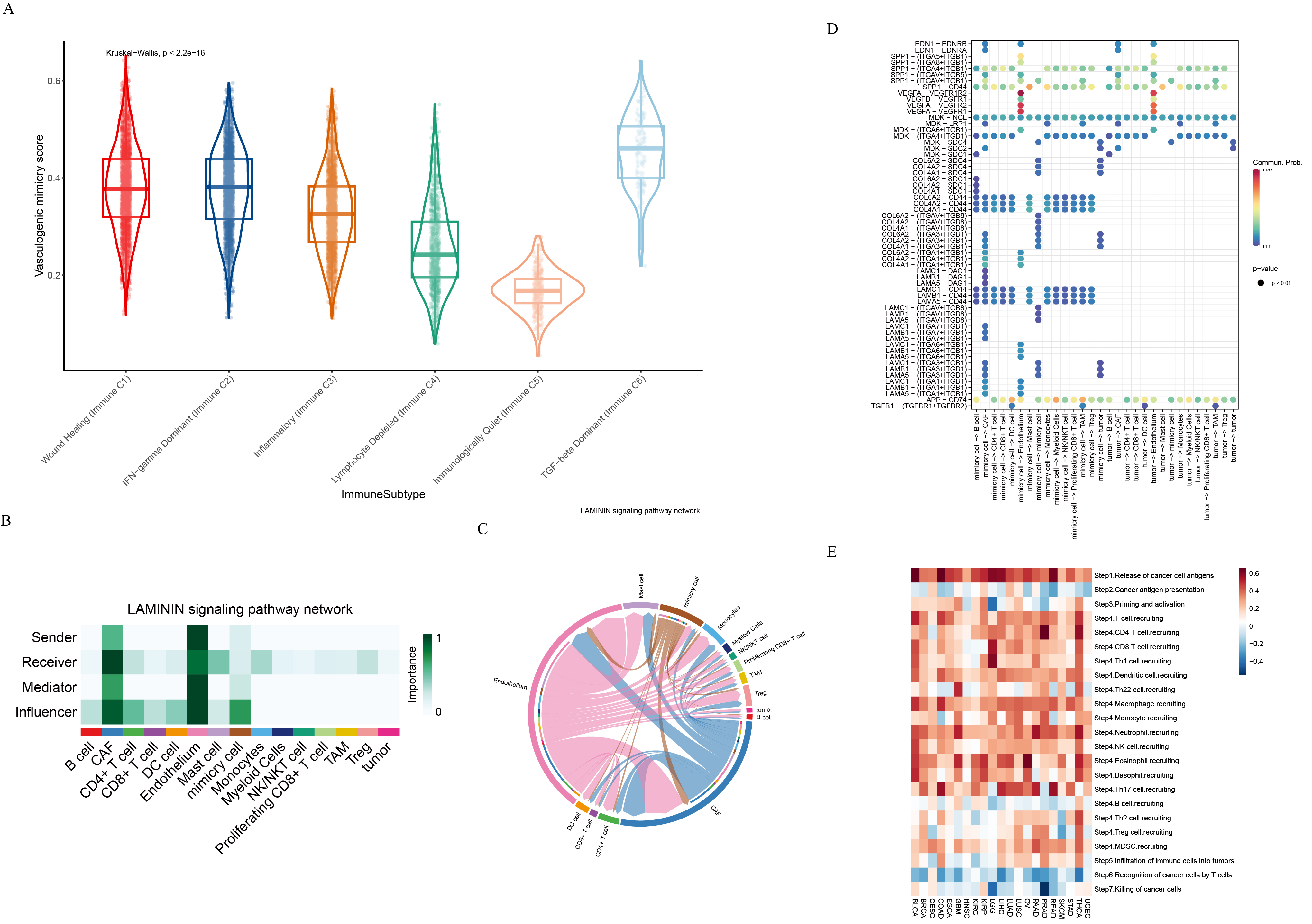

Supplement: Supplementary file 8 [file Image5.JPEG]

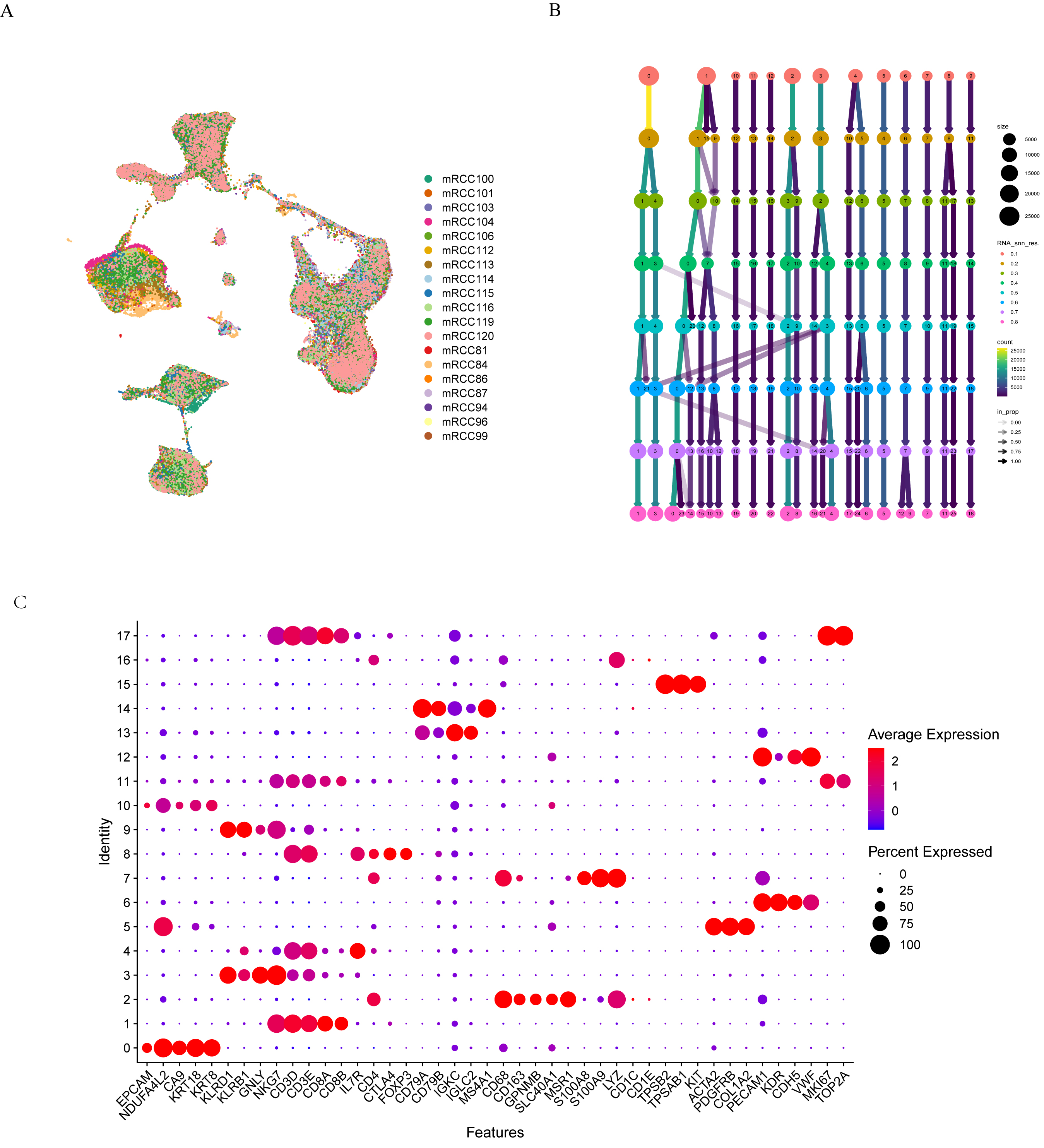

Supplement: Supplementary file 9 [file Image6.JPEG]
